# Cobaviruses – a new globally distributed phage group infecting *Rhodobacteraceae* in marine ecosystems

**DOI:** 10.1038/s41396-019-0362-7

**Published:** 2019-02-04

**Authors:** Vera Bischoff, Boyke Bunk, Jan P. Meier-Kolthoff, Cathrin Spröer, Anja Poehlein, Marco Dogs, Mary Nguyen, Jörn Petersen, Rolf Daniel, Jörg Overmann, Markus Göker, Meinhard Simon, Thorsten Brinkhoff, Cristina Moraru

**Affiliations:** 1 Institute for Chemistry and Biology of the Marine Environment, University of Oldenburg, Carl-von-Ossietzky-Str. 9 -11, D-26111 Oldenburg, Germany; 2 Leibniz-Institut DSMZ—Deutsche Sammlung von Mikroorganismen und Zellkulturen GmbH, Inhoffenstraße 7 B, D-38124 Braunschweig, Germany; 3 Georg-August-University Göttingen, Institute of Microbiology and Genetics, Department of Genomic and Applied Microbiology & Göttingen Genomics Laboratory, Grisebachstr. 8, D-37077 Göttingen, Germany; 4 Centre for Marine Bio-Innovation, School of Biological, Earth and Environmental Sciences, The University of New South Wales, Kensington, NSW 2052 Australia

**Keywords:** Water microbiology, Virology, Water microbiology, Virology

## Abstract

Bacteriophages are widely considered to influence bacterial communities, however most phages are still unknown or not studied well enough to understand their ecological roles. We have isolated two phages infecting *Lentibacter* sp. SH36, affiliated with the marine *Roseobacter* group, and retrieved similar phage genomes from publicly available metagenomics databases. Phylogenetic analysis placed the new phages within the Cobavirus group, in the here newly proposed genus Siovirus and subfamily Riovirinae of the *Podoviridae*. Gene composition and presence of direct terminal repeats in cultivated cobaviruses point toward a genome replication and packaging strategy similar to the T7 phage. Investigation of the genomes suggests that viral lysis of the cell proceeds via the canonical holin-endolysin pathway. Cobaviral hosts include members of the genera *Lentibacter*, *Sulfitobacter* and *Celeribacter* of the *Roseobacter* group within the family *Rhodobacteraceae* (*Alphaproteobacteria*). Screening more than 5,000 marine metagenomes, we found cobaviruses worldwide from temperate to tropical waters, in the euphotic zone, mainly in bays and estuaries, but also in the open ocean. The presence of cobaviruses in protist metagenomes as well as the phylogenetic neighborhood of cobaviruses in glutaredoxin and ribonucleotide reductase trees suggest that cobaviruses could infect bacteria associated with phototrophic or grazing protists. With this study, we expand the understanding of the phylogeny, classification, genomic organization, biogeography and ecology of this phage group infecting marine *Rhodobacteraceae*.

## Introduction

Viruses infecting bacteria and archaea are abundant in marine systems and are major drivers of biogeochemical cycles, through their ability to lyse cells, express auxiliary metabolic genes (AMG) and mediate horizontal gene transfer [1–3]. Marine habitats contain abundant and extremely diverse viral communities, the majority of which remains uncultivated [4–6].

Marine *Rhodobacteraceae* are an important group of heterotrophic bacteria in the marine environment. They are known under the operational term “*Roseobacter* group” [7] and their phages are known as roseophages. Marine *Rhodobacteraceae* show high metabolic versatility, metabolize a wide variety of organic compounds, degrade dimethylsulfoniopropionate, perform anoxygenic photosynthesis and produce various secondary metabolites [7–9]. *Rhodobacteraceae* are present in diverse marine habitats (pelagic, sediment, surface-associated) and are most abundant in coastal temperate to polar regions [10–13]. Members of *Rhodobacteraceae* typically increase in abundance during algal blooms, where they dominate the active bacterial community, utilize algal exudates and lysates and potentially engage in both mutualistic and pathogenic interactions with the algae [14–18]. Amongst the roseobacters, *Lentibacter* is a genus relevant in coastal and estuarine waters, where its relative abundance can be up to 30% of the bacterial community [19], and has been repeatedly isolated from algal blooms in different geographical locations [20, 21].

In contrast to the large diversity of marine *Rhodobacteraceae*, which includes currently over 70 genera [22], only a few roseophages, infecting seven genera, have been isolated so far. More than half of the cultivated roseophages belong to the N4-like phage group [23–28] and recent taxonomic reevaluation suggests that they likely form a subfamily within *Podoviridae* [29]. Other roseophages have been classified in the *Podoviridae* or *Siphoviridae* families [30–38].

The SIO1 phage infecting *Roseobacter* sp. SIO67 was the first described roseophage and the first sequenced marine phage [30]. Highly similar phages (>96% nucleotide identity) were isolated twelve years later from the same geographic area, providing evidence that marine viruses can exist as discrete populations over long periods of time [39]. The SIO1 phage belongs to the *Podoviridae* and is distantly related to the T7 phage [30], but has no RNA polymerase. A close relative of SIO1 is the roseophage P12053L infecting *Celeribacter marinus* IMCC12053 [33, 40]. Hardies et al. [41] assigned SIO1 and *Vibrio* phage VpV262 to a T7 supergroup. This supergroup includes T7 related phages with an RNA polymerase (the *Autographivirinae* subfamily), and also phages without the RNA polymerase. Hardies et al. [40] showed that all podoviruses, except those in the *Picovirinae* subfamily, have a tail structure related to T7 and propose their placement within the transient tail structural homology group, with the following subgroups: the T7 subgroup (the *Autographivirinae*), the epsilon15 subgroup, the phage RIO-1 subgroup (including SIO1 phage), the P22 subgroup, the N4 subgroup, and a new subgroup represented by *Pseudomonas* phage F116.

In this study, two novel roseophage species infecting *Lentibacter* sp. SH36 were isolated and sequenced. The recruitment from sequence data sets of similar genomes, including that of SIO1 and of environmental phages, has led to the delineation of a new group of phages which we termed the “Cobavirus” group. Furthermore, we carried out a comprehensive analysis of the phylogeny, classification, genomic organization, host range and ecology of the Cobavirus group, infecting also two other roseobacter genera. A survey of its global biogeography showed that cobaviruses occur mainly in coastal but also in oceanic water masses from temperate to tropical oceans.

## Materials and methods

### Phage enrichments and isolation of ICBM1 and ICBM2 phages

Surface seawater was collected from multiple stations (53.978 8.059; 53.937 7.806; 53.896 7.535; 53.840 7.255; 53.793 6.997) in the southern North Sea, during a phytoplankton bloom in March 2015, on board of the cruise ship RV Heincke. Further, the seawater from each station was filtered on board through 0.7 µm filters, 47 mm in diameter (GTTP filters, Millipore). To prevent clogging, the filters were exchanged every 2 liters. The seawater from all stations was pooled, transported to the laboratory and stored at 4 °C in the dark. Two phage enrichments (S1 and S2), each of 100 ml, were set up by mixing 90 ml of freshly filtered (Nalgene rapid-flow, 0.2 µm, PES membrane, ThermoScientific) seawater with 10 ml of 10x ASW (see SI file S1 text) and 2.1 ml of exponentially growing host culture *Lentibacter* sp. SH36 (final OD_600_ = 0.006). Two controls were prepared in parallel. The first, a positive control (PC) for host growth, consisted of 100 ml 1x ASW and 2.1 ml of exponentially growing host culture (final OD_600_ = 0.006). The second, a negative control (NC) for growth of seawater bacteria contaminants, which might have passed through the 0.2 µm filter, consisted of 90 ml freshly filtered (0.2 µm) seawater and 10 ml 10x ASW. Bacterial growth was monitored by measuring the optical density at 600 nm (Beckmann DU520, USA). The cultures were incubated at 20 °C and 100 rpm overnight, until the enrichment cultures showed signs of cell lysis. Lysis was indicated by decreasing OD in S1 and S2 cultures compared to the positive control and by the presence of cell debris in S1 and S2. The cell free phage lysates from the S1 and S2 enrichments were used to isolate the ICBM1 and ICBM2 phages, respectively, by plaque assays and single plaque picking. To determine the host range, 94 strains (SI file S1 Table S1) covering the phylogenetic diversity of *Rhodobacteraceae* were challenged with ICBM1 and ICBM2 phages, at three different temperatures (15, 20, and 28 °C), using the spot and plaque assay. For long-term storage, the phages were infected into the host cells (before cell lysis), resuspended in glycerol and kept at −80 °C. Further details on phage isolation, host range determination and long-term storage procedures are found in SI file S1 text.

### ICBM1 and ICBM2 phage lysates

To obtain a high amount of ICBM1 and ICBM2 phage biomass for TEM and genome sequencing, two subsequent infection cultures of *Lentibacter* sp. SH36 with phage ICBM1 or ICBM2 were performed. For the first round of infection, 1x ASW was inoculated with exponentially growing *Lentibacter* sp. SH36 to a final OD_600_ of 0.006 and with phage ICBM1 or ICBM2 stock. After an overnight incubation at 20 °C and shaking at 100 rpm, lysis was observed, indicated by a decrease in the OD_600_ (in comparison with the control, non-infected culture) and cellular debris. The remaining cells and cell debris were removed by centrifugation (15 min, 4,000x*g*, 20 °C) and 0.22 µm filtration. For the second round of infection, a highly concentrated phage-host mixture was obtained by pelleting an exponentially growing culture of *Lentibacter sp. SH36* and re-suspending the cell pellet in the phage fraction from the first infection culture. After the phage-host mixture was incubated on ice for 15 min to facilitate phage absorption, an equal volume of 2x ASW was added to it and the infection culture incubated overnight at 20 °C and 100 rpm. After lysis, the phage fraction was obtained by centrifugation (15 min, 4,000x*g*, 20 °C) and 0.22 µm filtration to remove remaining cells and cell debris.

### Transmission electron microscopy

ICBM1 and ICBM2 phages were purified by CsCl_2_ centrifugation. Two staining procedures were performed for each phage prior to transmission electron microscopy (TEM): (1) ammonium molybdate staining and (2) uranyl acetate staining, see SI file S1 text for details. Phages negatively stained by uranyl acetate were used for capsid size measurements.

### Isolation and purification of phage DNA for sequencing

#### Phage isolates – extraction of DNA from virions

Phage DNA was extracted from cell free phage lysates obtained by infecting *Lentibacter* sp. SH36 with ICBM1 or ICBM2. The phage fraction was concentrated by polyethylene glycol precipitation, treated with DNase to remove free, chromosomal DNA, and then phage DNA was purified using the ChargeSwitch gDNA Mini Bacteria Kit (ThermoFisher Scientific). For details, see SI file S1 text.

#### Phage enrichments – extraction of DNA from the intracellular phage fraction

**Fig. 1:**
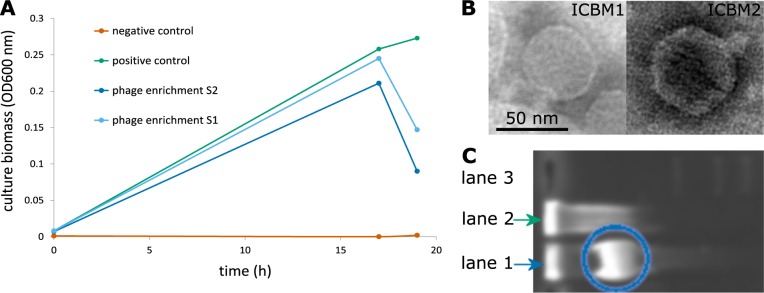
**a.** Enrichment of phages specific for *Lentibacter* sp. SH36 from North Sea water. The experimental setup consists of: (i) Phage enrichments (blue lines) – logarithmic phase cells added to nutrient amended, 0.2 µm filtered seawater, (ii) Positive control for cell growth (green line) – logarithmic phase cells added to artificial seawater and (iii) Negative control (orange line) – only nutrient amended, 0.2 µm filtered seawater, no cells added. In the S1 and S2 enrichments the decrease in OD at 18 h is most likely due to phage cell lysis. No growth was detected in the negative control. **b**. Transmission electron micrograph of molybdenum stained, cell debris bound Lentibacter virus vB_LenP_ICBM1 and uranyl acetate stained, free Lentibacter virus vB_LenP_ICBM2. Scale bar: 100 nm. **c**. Agarose gel electrophoresis of cellular DNA from i) *Lentibacter* sp. SH36 phage infected cells, S2 enrichment (lane 1, blue arrow) and ii) not infected cells, positive control for cell growth (lane 2, green arrow). Blue circle: intracellular phage DNA. Lane 3: 1 kbp Plus DNA Ladder

Cells from the S1 and S2 enrichments and from the PC culture were collected when cell lysis was detected in S1 and S2. As expected, the NC culture showed no growth. Cells were embedded in agarose, digested to remove the proteins and lipids, and the phage DNA was separated from the chromosomal DNA by agarose gel electrophoresis. The phage DNA fraction in S1 and S2 was identified by comparison with DNA from the PC culture (Fig. 1c), and phage DNA was extracted from the gel, purified and sequenced. For details, see SI file S1 text.

### Genome sequencing and assembly

The ICBM1 phage and the S2 enrichment were sequenced using both Illumina (paired-end technology 2 × 300 bp) and PacBio technologies. The ICBM2 phage and the S1 enrichment were sequenced only by Illumina. The Illumina and the PacBio assemblies were performed separately and they resulted in identical phage genomes (SI file S1 Table S2). Error free assembly of the PacBio samples was possible due to the high coverage obtained (>4,000x). The phage genomes are available in the NCBI GenBank database under the following accession numbers: MF431617 (ICBM1), MF431616 (ICBM2) and MF431615 (ICBM3, assembled from the S2 phage enrichment). For details about sequencing and assembly, see SI file S1 text.

### Retrieval of phage genomes related to ICBM1 and ICBM2

The following datasets were queried for sequences related to ICBM1 and ICBM2: (i) the Tara Ocean Viromes [4], (ii) the Earth Virome [5], (iii) the Global Ocean Virome [3], (iv) the IMG/VR [42] and (v) the Environmental Viral Genomes [6]. The Tara Oceans Viromes (assembled DNA contigs and predicted proteins) and Global Ocean Virome datasets were downloaded from the iVirus (http://ivirus.us/ [43],) using the CyVerse platform and its Discovery Environment (https://de.cyverse.org/de/ [44],). The Earth Virome dataset (assembled DNA contigs) was downloaded from http://portal.nersc.gov/dna/microbial/prokpubs/EarthVirome_DP/. The downloaded datasets were imported in Geneious 9.1.5, transformed in BLAST databases and queried by megaBLAST (e-value 1e^−5^), using the ICBM1 and ICBM2 genomes and by BLASTp (e-value 1e^−5^) using the portal and terminase proteins of the ICBM1, ICBM2, SIO1 (only the terminase protein was used) and P12053L phages. The IMG-VR viral sequence database was queried by BLASTn (e-value 1e^−5^) webservice offered at https://img.jgi.doe.gov/cgi-bin/vr/main.cgi, using the ICBM1 and ICBM2 genomes. The proteins retrieved by Blastp were added to the databases of terminase or portal proteins from known phages, followed by multiple alignment with Muscle and calculation of phylogenetic trees with FastTree v 2.1.5 [45]. Further, the proteins in the vicinity of ICBM1, ICBM2, SIO1 and P12053L were selected, and their corresponding contigs retrieved. These contigs were pooled with all those retrieved by nucleotide Blast. All contigs smaller than 34 kbps (~85% of the ICBM1 genome length) were considered incomplete and removed.

### Genome-based phylogeny and classification

To reconstruct the whole genome-based phage phylogenetic tree, a set of genomes comprising all podoviral genomes recognized by the International Committee of Taxonomy of Viruses (ICTV) was supplemented with the cobavirus-related genomes retrieved from the different public sequence datasets (see above). For consistency, open reading frames (ORFs) for the complete set of genomes were detected using MetaGeneAnnotator [46], which was implemented in the VirSorter program [47]. Using the Virus Classification and Tree Building Online Resource (VICTOR [48], available at https://victor.dsmz.de), all pairwise phage comparisons of the amino acid sequences were conducted via the underlying Genome-BLAST Distance Phylogeny (GBDP) method [49] under settings recommended for prokaryotic viruses [48]. The resulting intergenomic distances (including 100 replicates each) were used to infer a balanced minimum evolution tree with branch support via FASTME including SPR postprocessing [50] for each of the formulas *d*_0_, *d*_4_, and *d*_6_. The trees were rooted at the midpoint [51] and visualized with iTOL [52]. Taxon boundaries at the species, genus, subfamily and family level were estimated with the OPTSIL program [53] using the recommended clustering thresholds [49] and an *F* value (fraction of links required for cluster fusion) of 0.5 [54].

### Genome annotation and protein clustering

All phage genomes compared in this study, including the already published ones, were re-annotated using the same procedure to eliminate differences resulted from different annotation pipelines. Initially, ORFs were detected using MetaGeneAnnotator [46] implemented in VirSorter [47]. Proteins were then annotated by comparing them with several databases and manually deciding the final annotations. The NR database (http://ncbi.nlm.nih.gov/) was queried using Protein-Protein BLAST 2.6.0+, the InterPro database v66.0 [55] was queried using InterProScan 5.27-66.0 tool [56], and the prokaryotic viruses orthologous groups database [57] was queried using hmmscan command from HMMER 3.1b2 package [58]. The proteins were clustered by first performing an all against all BlastP, with an e-value threshold of 1e^−5^ and a bitscore threshold of 50, and the results were inputted into the mcl program, with the parameters “-I 2 --abc”. The online tool tRNAscan-SE v. 2.0 (http://lowelab.ucsc.edu/tRNAscan-SE/index.html [59],) was used for tRNA prediction. Rho-independent terminators were predicted with ARNold http://rna.igmors.u-psud.fr/toolbox/arnold/index.php. Only the terminators with deltaG higher than 10.5 were considered. Details of genome features, protein clusters and DNA sequences for all cobaviruses identified are listed in SI file S2 and S3. The comparative genome map was generated using the genoPlotR package [60] from the R programming environment (https://www.r-project.org/).

### Phylogenetic analyses of single proteins

Phylogenetic trees were constructed for the terminase protein, to gain insights about the genome ends. Phylogenetic analysis of spanin, glutaredoxin and cobalamin dependent ribonucleotide reductase (RNR) proteins was conducted to find insights about the hosts of the environmental cobaviruses and their habitat. Proteins were aligned with Muscle, and then phylogenetic trees were constructed using the FastTree v 2.1.5 program [45] integrated as a plugin in Geneious v 9.1.5 (http://www.geneious.com [61]), using default parameters. Phylogenetic trees were visualized using FigTree v1.4.3. ([62], available at http://tree.bio.ed.ac.uk/software/figtree/).

### Phylogenetic analysis of the host rRNA

The 16 S rRNA gene phylogenetic tree was constructed using the ARB software package (www.arb-home.de/ version arb-6.0.2) [63]. Sequences of the type material (>1,300 bp) were used for the backbone-tree using the neighbor joining method with 1500 replicates. Shorter sequences used in this study were added afterwards by parsimony interactive without using a filter.

### Biogeographic distribution of cobaviruses and read mapping

#### Metagenomic data sets, download and preprocessing

The unassembled datasets used for read mapping were downloaded from the European Nucleotide Archive (ENA, https://www.ebi.ac.uk/ena). A complete list of datasets used is given in SI file S4. The Tara Ocean survey datasets have been cleaned before their deposition at ENA [64], and thus, we used them as such for read mapping. We cleaned the remaining datasets using BBDuk from the BBTools package (BBTools (https://jgi.doe.gov/data-and-tools/bbtools/), as follows: (i) reads corresponding to the Enterobacteria phage phiX174 were filtered out; (ii) sequences of Illumina adapters and primers as provided in the BBTools package were removed (ktrim = r k = 21 rcomp = t mink = 11 hdist = 1 tpe tbo); (iii) low quality (quality value lower than 20) nucleotides from both read ends were removed and reads with low average quality (<20) or short length (<30 bases) were also removed (qtrim = rl trimq = 20 ftm = 5 maq = 20 minlen = 30). Quality control of the cleaned samples was performed on a subset of random samples, using FastaQC. The metadata associated with the metagenomes were retrieved from the NCBI site, BioSamples databases. In specific cases, if metadata were missing, we received them by direct contact with the principle investigators for the respective projects.

#### Read mapping

BBMap from the BBTools package was used to map the reads from the unassembled datasets to the cobaviral genomes. The output was sent to Samtools View and then to Samtools Sort to produce a sorted bam file. A phage was considered to be present in a particular sample when at least 75% of its genome was covered by reads with at least 90% identity, as previously determined [65]. The relative abundance of a phage genome in a sample was calculated by the following formula: “number of bases at ≥90% identity aligning to the genome / genome size in bases / library size in gigabases (Gb)”. All code used for read mapping and data analysis are available in SI files S7a-d.

## Results and discussion

### Isolation and host range of two *Lentibacter* sp. SH36 viruses

Two strictly lytic bacteriophages, Lentibacter virus vB_LenP_ICBM1 (ICBM1) and Lentibacter virus vB_LenP_ICBM2 (ICBM2) were isolated to pure cultures from phage enrichments S1 and S2, respectively (Fig. 1a). The phage source in the enrichments was surface seawater collected during a March 2015 algal bloom in the southern North Sea. The host was *Lentibacter* sp. SH36, which was isolated from a seawater sample taken on 12 May 2007 in the southern North Sea during a phytoplankton bloom [21]. ICBM1 and ICBM2 phages negatively stained with uranyl acetate had isometric capsids with hexagonal cross-sections of 58.7 ± 3.7 nm (sample size = 100 phages) and 59.2 ± 2.8 nm (sample size = 100 phages), respectively, and short tails (Fig. 1b). Assessment on 94 *Rhodobacteraceae* strains (SI file S1 Table S1) showed that ICBM1 and ICBM2 have a narrow host range, infecting only *Lentibacter* sp. SH36.

### Sequencing the phage isolates and enrichments

To gain insights into the diversity of the phage enrichments, we sequenced both the purified phages (ICBM1 and ICBM2) and the intracellular phage fraction of the enrichments (Fig. 1c, SI file S1 Table S2).

**Fig. 2:**
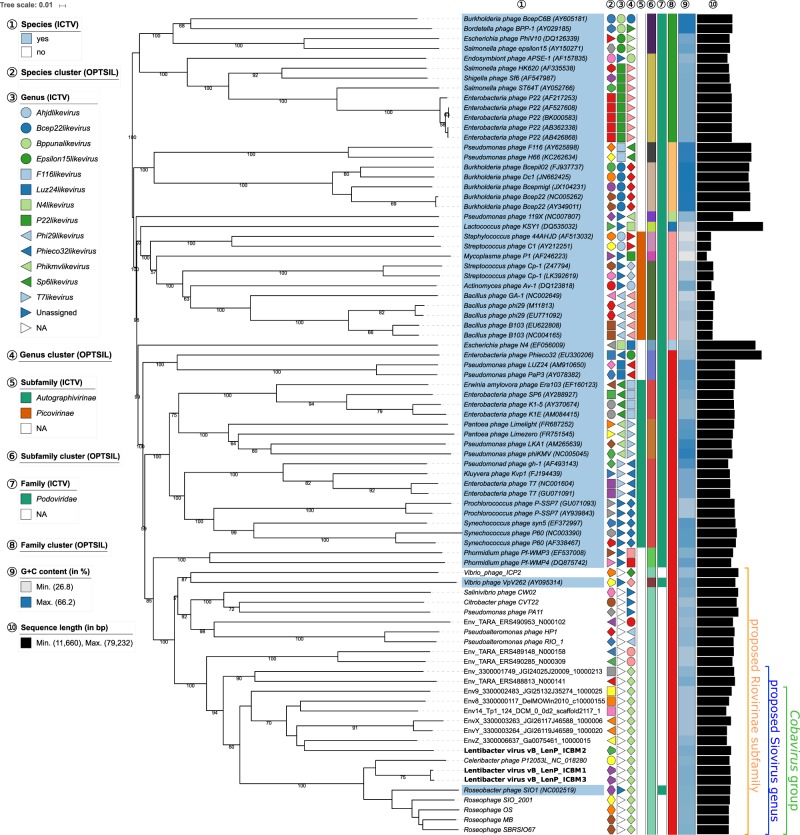
Phylogenetic positioning of the *Lentibacter* sp. SH36 viruses and their relatives within the *Podoviridae*. The whole-genome-based phylogeny was inferred using the Genome-BLAST Distance Phylogeny method implemented in the VICTOR web service, using the amino acid data. Internal branch labels represent pseudo bootstrap support values if larger than 50%. The proposed subfamily Riovirinae, the proposed genus Siovirus and the Cobavirus group (sioviruses with cobalamin-dependent RNR) are annotated at the right-hand side. Further information regarding the affiliation of phages to ICTV taxa and OPTSIL clusters as well as G + C content and genome sizes is described within the figure legend (circled numbers). “Viruses annotated as “Unassigned” in legend “Genus (ICTV)” have been assigned to both an ICTV species and family but not to a genus level, whereas “NA” refers to viruses which have not been recognized as a taxa by the ICTV. The affiliation of one or more viruses to a distinct species, genus, subfamily or family cluster is indicated by a specific symbol of same shape and color

From the S2 enrichment two complete phage genomes were assembled, that of ICBM2 and of a third phage. The latter had 99.6% sequence similarity at nucleotide level with ICBM1 (under VICTOR formula *d*_0_ see SI file S6) and was named Lentibacter virus vB_LenP_ICBM3 (ICBM3). Both ICBM1 and ICBM3 have been assembled twice, once from Illumina and once from PacBio reads, with identical results (SI file S1 Table S2). Therefore, differences between them were real and not due to sequencing errors. According to the VICTOR [48] results, ICBM1 and ICBM3 formed a species cluster, whereas ICBM2 represented a distinct species (see section below and Fig. 2).

From the S1 enrichment we retrieved an ICBM3-like genome (99.9% identical with ICBM3, differences potentially due to sequencing errors, see SI file S1 Figures S1 and S2). Read mapping with a cutoff of 100% read identity showed that both ICBM1 and ICBM3 were present in S1 (Table 1, SI file S1 Figure S2). The presence of both ICBM1 and ICBM3 phages in the S1 enrichment is strengthened by the isolation of ICBM1 from this enrichment and it indicates microdiversity. Microdiversity in phage enrichments have been previously reported [66] and it potentially reflects the situation in the original seawater.

**Table 1:** Abundance of cobaviruses in the S1 and S2 phage enrichments

Enrichments		ICBM1				ICBM2				ICBM3			
Name	Mbps	95% read identity		100% read identity		95% read identity		100% read identity		95% read identity		100% read identity	
		% genome covered	Abundance^a^	% genome covered	Abundance	% genome covered	Abundance	% genome covered	Abundance	% genome covered	Abundance	% genome covered	Abundance
S1	232.2	100.0	95.3	100.0	54.1	5.3	n.d.	0.0	0.0	100.0	97.5	100.0	66.7
S2	472.6	100.0	56.2	96.8	26.9	100.0	39.7	100.0	28.1	100.0	58.9	100.0	41.4

^a^Abundance expressed in % from total bases

Using a 95% read identity cutoff for mapping, all reads in the enrichments recruited either to the ICBM1/ICBM3 or to the ICBM2 genomes (Table 1). This indicates that, without considering microdiversity, most likely no other phage was present and our isolation efforts retrieved the complete phage diversity in the enrichments at the species level.

### Retrieval of similar phage genomes and phylogenetic positioning

Cultivated and environmental phage genomes similar to ICBM1 and ICBM2 were found in public sequence data sets. The related cultivated phages were P12053L infecting *Celeribacter marinu*s IMCC12053, SIO1 infecting *Roseobacter* sp. SIO67 and four other SIO1 related strains, infecting *Roseobacter* sp. SIO67 and *Roseobacter* sp. GAI-101 [39]. From the environmental genomes, only those bigger than 35 kbp were considered for further analysis (see Table 2 and SI file S1 text). These were JGI26117J46588_1000006, JGI26119J46589_1000020, Ga0075461_10000015, DelMOWin2010_c10000155, JGI25132J35274_1000025, Tp1_124_DCM_0_0d2_scaffold2117_1, which we chose to identify in this manuscript as EnvX, EnvY, EnvZ, Env8, Env9 and Env14, respectively.

**Table 2:** Environmental cobaviruses, retrieval from databases

Genome	Datasets	(meta)genome accession in IMG/VR / GOV datasets	Contig name in IMG/VR / GOV datasets.	NCBI BioProject	NCBI BioSample	NCBI Run	Coordinates	Location
EnvX	IMG/VR	3300003263	JGI26117J46588_1000006	PRJNA366974	SAMN06267889	SRR5268549	36.25 N 122.2099 W	Monterey Bay
EnvY	IMG/VR	3300003264	JGI26119J46589_1000020	PRJNA366976	SAMN06267891	SRR5268667	36.25 N 122.2099 W	Monterey Bay
EnvZ	IMG/VR	3300006637	Ga0075461_10000015	PRJNA375611	SAMN06343913	SRR5600317	39.283 N 75.3633 W	Delaware Bay
Env8	IMG/VR	3300000117	DelMOWin2010_c10000155	PRJNA336873	SAMN05518585	missing	39.0042816 N 77.1012173 W	Delaware Coast
Env9	IMG/VR	3300002483	JGI25132J35274_1000025	PRJNA366059	SAMN06268330	SRR5251700	18.9200 N 104.8900 W	Pacific Coast of Mexico
Env14	GOV	124_MIX	Tp1_124_DCM_0_0d2_scaffold2117_1	PRJEB4419	TARA_R100000700	ERR599367	-9.0714 N-140.5973 E	Marquesas Islands

The VICTOR method [48] for phage phylogeny and classification was used because it is universal and allows for an informed decision on the evolutionary relationships between prokaryotic viruses. The method was thoroughly optimized against a large reference dataset of genome-sequenced taxa recognized by the International Committee on Taxonomy of Viruses (ICTV) and showed a high agreement with the classification, particularly at the species and genus level.

The genome-based VICTOR [48] phylogeny combined with taxon boundaries prediction based on OPTISIL [53] showed that the *Lentibacter* sp. SH36 phages (ICBM1, ICBM2 and ICBM3), together with SIO1, P12053L and some of the environmental genomes formed a highly supported genus level clade (Fig. 2). This proposed genus was tentatively named here as Siovirus (from the SIO1 phage) (Fig. 2). Most of the sioviruses had a class II, cobalamin dependent RNR and were placed within one cluster, which we called the Cobavirus (cobalamin-dependent) group. Two of the environmental sioviruses had a class I RNR and formed a separate clade. RNRs are used to convert host ribonucleotides in deoxyribonucleotides necessary for phage replication. Because the RNR class is predictive of the phage habitat [67] and class II RNRs point toward an association with phototrophic protists, we focused further on the Cobavirus group, which included all cultivated and part of the environmental sioviruses (Fig. 2).

In agreement with previous findings for the SIO1 and P12053L phages [40], the cobaviruses clustered within the RIO-1 subgroup (Fig. 2). The OPTISIL based taxon boundaries reported by VICTOR [48] suggested that the RIO-1 subgroup forms a maximally supported group, which we propose to define as a new subfamily in the *Podoviridae*, and tentatively named here Riovirinae (from the RIO-1 phage).

We have excluded the ICBM3 phage from further analysis, due to its high similarity with ICBM1 (Fig. 2 and SI file S1 Figure S6) and the phages SIO1_2001, OS, MB, SBRSIO67, because their genomes contained several regions of sequence uncertainty (long stretches of Ns).

### Genomic organization

#### Genome termini

Within the proposed subfamily Riovirinae, the genome ends of the VpV262 and RIO-1 phages have been characterized and consist of direct terminal repeats (DTRs) [41, 68]. The SIO1 phage was reported to have inverted repeats [30], and no information was available for P12053L. We used coverage information and read structure from genome sequencing to determine the termini of the ICBM1, ICBM2 and ICBM3 phages. The Illumina reads were not informative regarding the genomic ends, as expected for sequencing libraries prepared with the NexteraXT kit [69]. On the other hand, the PacBio reads clearly showed the presence of short DTRs (159–173 bp) at the genome ends of all three phages (SI file S1 text).

We further investigated the ends of the other cobaviral genomes. The ends of SIO1 were originally determined after whole genome sequencing through a combination of shotgun cloning and Sanger technology. Inverted repeats (251 to 637 bases) detected at the ends were presumed to be involved in replication [30]. Our own analysis indicated that the ends were most likely placed incorrectly, probably due to low read coverage. Several facts supported our conclusion. First, in phylogenetic trees for the terminase gene, the phages ICBM1, ICBM3 and SIO1 grouped closely (SI file S1 Figure S7), indicating that they likely have similar genome packaging strategies [70]. Second, re-sequencing of the SIO1 genome did not retrieve the complete region of the inverted repeats [39]. This was initially attributed to difficulties in PCR amplification of the repeats. On the other hand, the lack of retrieval can also suggest misassembly of the original genome sequence in this region. Third, from the three inverted repeats, none were placed at the exact ends of the genome and two of the inversions were located at the same end (SI file S1 Figure S8). Inverted terminal repeats at the genome ends are found in viruses which replicate by a protein-primed mechanism, where they are positioned at the exact ends of the genomes [71, 72]. Hence, it is unlikely that the three inverted repeats of the SIO1 phage have a role in replication. Fourth, in its original order the SIO1 genome shows an ORF free region exactly in between two gene modules (SI file S1 Figure S8), a region which shares high sequence similarity (~80% identity) with the DTRs from ICBM1 and ICBM3 phages (SI file S1 Figure S9). An inspection of podovirus genomes from public databases revealed that related phages can have DTRs with a nucleotide identity within the 70–100% range (SI file S1 Table S5). Therefore, we used the DTRs from ICBM1 to find the genome termini and rearrange the gene order accordingly, not only for the SIO1 phage, but also for the P12053L and environmental cobaviruses (SI file S1 text).

Our results show that the cultivated cobaviruses have DTRs. The presence of DTRs indicate that cultivated cobaviruses, similar to the T7 phage, most likely use long concatemeric DNA molecules as intermediates in replication and packaging, concatemers formed by the annealing of 3′ single strands resulted at the DTR level during replication [73, 74]. The 5′ ends of all cobaviral DTRs have a conserved, G + C rich region (SI file S1 Figure S9), underlining a potentially more important role of this region in genome circularization or replication, for example as enzyme binding site. The phylogenetic positioning in the GBDP-based VICTOR tree (Fig. 2) as well as in the terminase tree (SI file S1 Figure S7) suggests that the environmental cobaviruses also have DTRs and thus potentially the same DNA replication strategy.

#### Gene composition and modular organization

**Fig. 3:**
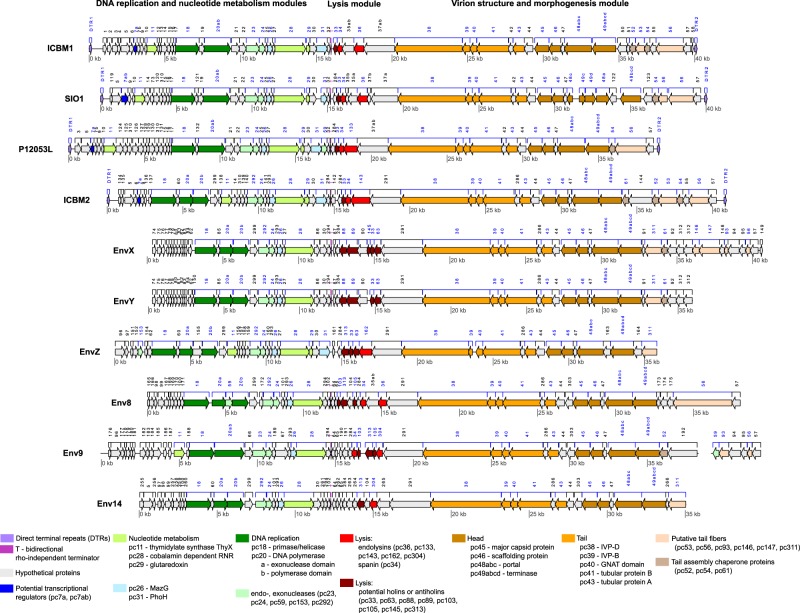
Genome map of cultured and environmental Cobaviruses. The genomes are centered in the bidirectional rho-independent terminator. With the exception of EnvX and Env9, all other environmental genomes are incomplete, with sequence information missing at the two ends of the genomes (the host interaction and tail fibers modules). Blue numbers indicate protein clusters with functional annotation

ICBM1 and ICBM2 phages had linear genomes of ~40 kb, a G + C content of ~47% (SI file S1 Table S3) and 58 and 55 ORFs, respectively. More than half of the ORFS coded for hypothetical proteins. No tRNAs were found. The genes were organized in two genomic arms, with opposite transcriptional directions and separated by a bidirectional, rho-independent transcriptional terminator (Fig. 3 and SI file S1 Table S4). We found protein-encoding genes for replication and nucleotide metabolism on the left arm: two nucleases, a DNA polymerase, a dual primase/helicase, a cobalamin dependent RNR, a glutaredoxin, a ThyX thymidylate synthase (ThyX), a guanosine 3′, 5′-bispyrophosphate (ppGpp) hydrolase (MazG) and a P-loop containing nucleoside triphosphate hydrolase (PhoH) [75–80]. On the right genomic arm, we found genes for lysis and virion structure and morphology. Both phages had spanins, which were easy to recognize due to their specific architecture. At the N terminus the spanins had a lipoprotein domain for binding to the outer membrane. At the C terminus they had a transmembrane domain for binding to the inner membrane [81]. For endolysins, ICBM1 had a lysozyme-like protein and ICBM2 had an N-acetylmuramoyl-L-alanine amidase. The lysis genes were followed by genes for the internal virion proteins (IVP) B and D, a Gcn5-related N-acetyltransferase (GNAT), the tubular proteins A and B, a major capsid protein, a scaffolding protein, a portal protein, a large terminase subunit, two tail fibers and three tail assembly chaperone proteins (see Fig. 3). With the exception of the endolysins, all other genes have been previously annotated in SIO1 or P12053L phages [30, 33, 39, 40, 81]. A previous study [82] annotated the gene for the pc40 protein from SIO1 as a long-chain fatty acid transporter (FadL) and thus listed it as AMG. However, our BlastP and InterProScan searches identified pc40 as a Gcn5-related N-acetyltransferases (GNAT). Based on the GNAT domain, pc40 could correspond to gp13 from T7, which is also positioned next to the internal virion proteins and has been suggested to play a role in virion morphogenesis [83].

Within the Cobavirus group, the genetic composition and synteny was mostly conserved (see Fig. 3). All genomes were organized in two arms, with genes for replication and nucleotide metabolism on the left and lysis and virion structure and morphogenesis on the right. This genomic organization was not previously reported for the SIO1 and P12053L phages, but it became evident once the genomes were rearranged according to the DTR positions (see section above and SI file S1 text). Furthermore, it appears in other members of the proposed Riovirinae subfamily, although the contained modules can vary [68, 84]. Most cobaviruses had a bidirectional, rho-independent transcriptional terminator in between the two genomic arms, indicating a likely transcriptional separation (see Fig. 3). This type of terminator was shown to be functional in vitro for the Pf-WMP3 phage [85].

Most of the genes with a functional annotation in ICBM1 and ICBM2 phage were also found in all other cobaviruses, with the exception of glutaredoxin, ThyX and PhoH, which were not found in some of the environmental cobaviruses (see Fig. 3). The endolysins were found in all cobaviruses, with the exception of EnvX and EnvY. They were free of membrane anchoring domains, indicating that cell lysis most likely proceeds via the canonical holin-endolysin pathway [86, 87]. The endolysins were diverse both in sequence and enzymatic function, encoding either lysozyme-like domains, or N-acetylmuramoyl-L-alanine amidase or peptidase domains. The spanin was found in all cultured cobaviruses, and only in two of the environmental genomes, Env9 and Env8. In the vicinity of the spanin and endolysins genes we found several genes encoding one or two transmembrane domains, representing potential holins and antiholins (see Fig. 3).

In agreement with cobavirus phylogenetic positioning and virion morphology revealed by TEM (Fig. 1b), the genes present in the virion structure and morphogenesis module most likely indicated a podoviral, T7-like virion structure [88–90]. A conserved genetic composition and synteny characterized the genomic region between the lysis module and the terminase gene (Fig. 3). The genomic region between the terminase and the 3′ end of the genome was variable both in gene count and composition and it encoded the proteins required for tail fibers, fiber connectors or tail assembly proteins. Most proteins were unique to a single phage or shared by a few. Some proteins (pc53, pc56, pc311) were similar to tail fibers or fiber connectors from myoviruses or siphoviruses, as noticed for other phages in the RIO-1 subgroup [40]. For example, pc53 resembled the short tail fiber protein from the T4 phage, a myovirus [91]. The pc56 protein was similar with the L-shaped tail fiber protein from the T5 phage and the T5-like siphoviruses DT57C and DT571/2 [92]. Therefore, the tail fibers of the cobaviruses likely depart from the simplicity of T7-like fibers, which are formed from a single protein (gp17) directly connected to the tubular protein A.

### Cobaviral hosts

The Cobavirus group contained both cultivated phages, with known hosts, and environmental phages, with unknown hosts. To have an up to date phylogeny of the hosts of cultivated cobaviruses, we built a 16 S rRNA gene-based tree (SI file S1 Figure S10). Our results showed that *Roseobacter* sp. SI067 belongs to the *Lentibacter* genus (>99% nucleotide identity with the type species) and *Roseobacter* sp. GAI-101 to the *Sulfitobacter* genus (>98% nucleotide identity). Therefore, hosts of cultivated cobaviruses comprise members of the *Lentibacter, Sulfitobacter* and *Celeribacter* genera, within the *Rhodobacteraceae* family.

**Fig. 4:**
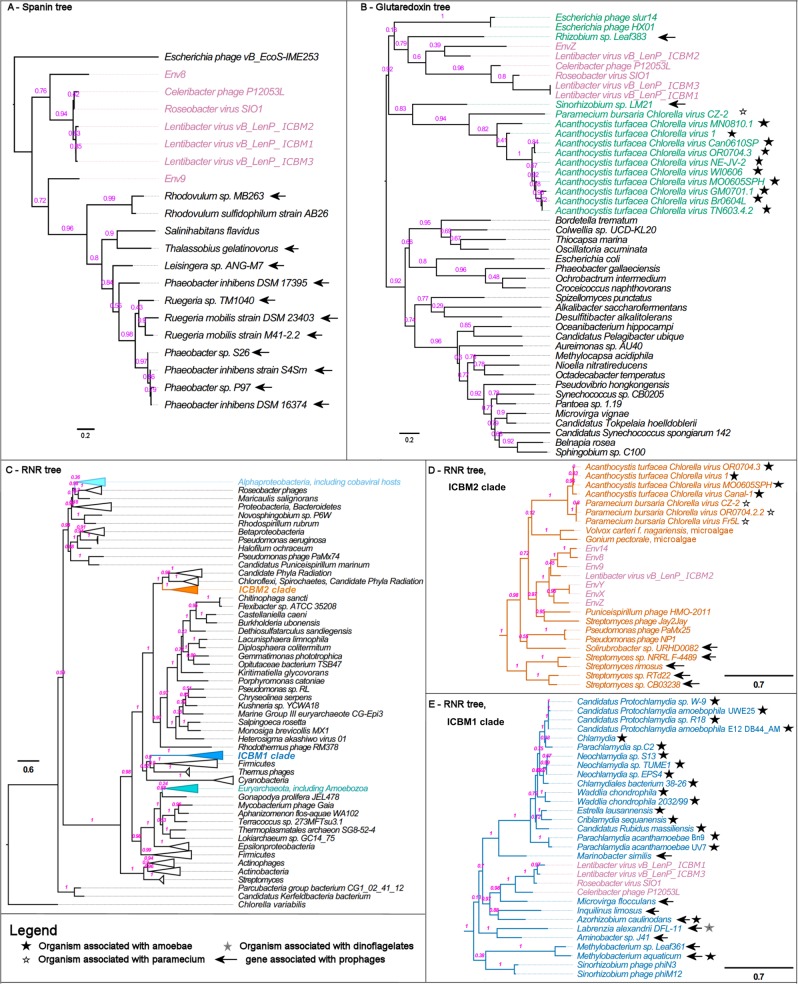
Phylogenetic analysis of the spanin (**a**), glutaredoxin (**b**) and ribonucleotide reductase (**c**–**e**) genes from cobaviruses. The evolutionary history was inferred using the approximately-maximum-likelihood method implemented in FastTree 2.1.5. The node labels represent Fast Tree support values. The tree is drawn to scale, with branch lengths measured in number of amino acid substitutions per site. Association of the bacteria to eukaryotic organisms is indicated by stars. Location of the spanin, glutaredoxin or RNR genes in prophage regions, predicted with PHASTER [94], is indicated by arrows and further detailed in SI file S1 Tables S6, S11 and S12

Furthermore, we searched for clues linking the environmental cobaviruses to potential hosts. A search in the CRISPR spacer database from IMG/VR returned no results, and no tRNAs where found within the cobavirus genomes. We found, however, three lines of evidence that point to environmental cobaviruses infecting members of the *Rhodobacteraceae* family. First, cobaviruses clustered into one genus, with nine out of 15 representatives known to infect *Rhodobacteraceae* members. According to Meier-Kolthoff and Göker [48], phage genera usually infect hosts within the same family. Second, all cobaviruses had a cobalamin-dependent RNR gene, encoding an enzyme used to reroute host resources toward phage replication. These phages need to infect bacteria able to synthesize cobalamin, and this ability is widespread within marine *Rhodobacteraceae* [93]. Genes involved in vitamin B12 synthesis are present in the two publicly available genomes from the cobaviral hosts. Additionally, in phylogenetic trees the RNRs from environmental cobaviruses clustered closely with ICBM2 (Fig. 4), whose host is *Lentibacter* sp. SH36. Third, all cultivated and two environmental cobaviruses (Env8 and Env9) had a spanin gene characteristic for roseophages. BLAST searches in the NR database from NCBI with the cobaviral spanins returned hits only from roseophages or members of *Rhodobacteraceae*, with the exception of one *E. coli* phage hit, which had very low similarity (Fig. 4a). This is not surprising, considering that spanins have little sequence homology to each other and Summer et al. [81] found no homolog for the SIO1 spanin. Using prophage prediction (PHASTER [94]), we determined that the spanins from *Rhodobacteraceae* genomes were present in putative prophage regions (SI file S1 Table S6). Therefore, phages infecting *Rhodobacteraceae* have similar spanins, another evidence that Env8 and Env9 most likely infect *Rhodobacteraceae*.

### Environmental distribution of the cobaviruses

Cobaviruses have been isolated from three distinct coastal locations in the Northern Hemisphere: SIO1 from the American coast of the Pacific Ocean (Scripps Pier, California) [30, 39], P12053L from the Yellow Sea, South Korea [33] and ICBM1 and ICBM2 from the North Sea, Germany (this study). Sequences related to the SIO1 and P12053L phages were previously reported in predominantly coastal viromes from the North Pacific USA coast (Scripps Pier, British Columbia), the Gulf of Mexico, the Arctic Ocean, the North Atlantic (Chesapeake Bay and Sargasso Sea) and the Yellow Sea (Goseong Bay) [39, 95–98].

**Fig. 5:**
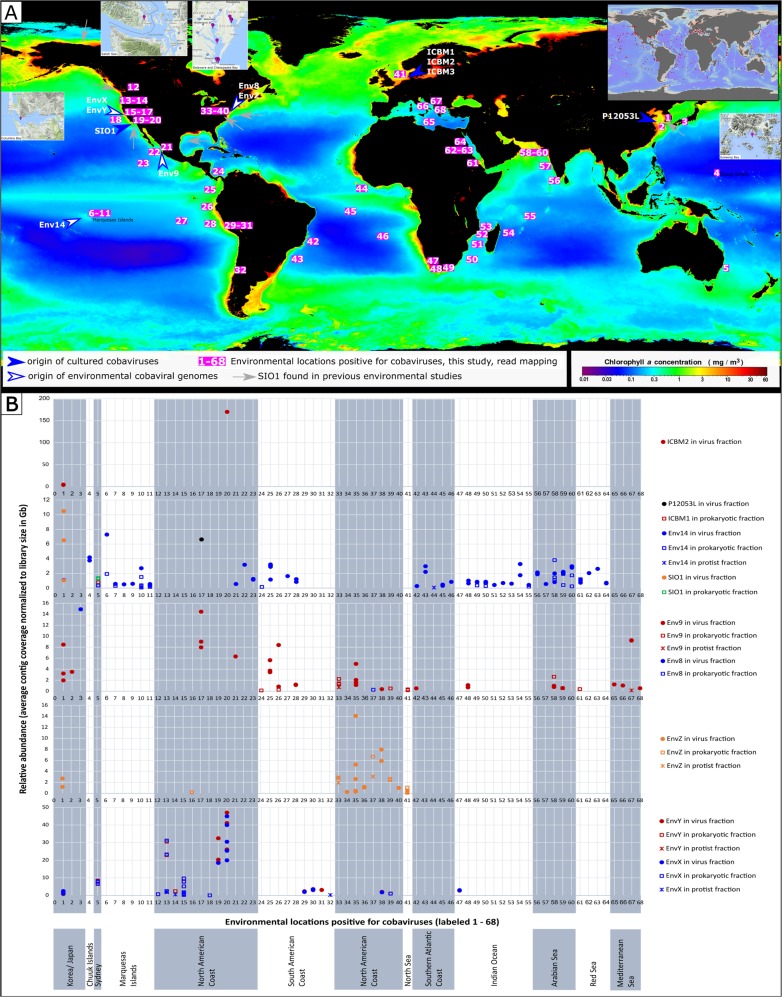
Global distribution of Cobaviruses (**a**) and their abundance (average contig coverage per Gb metagenome) in metagenomic samples from marine environments **(b**). **a** (i) main map – each location were cobaviruses were found by read mapping in this study is labeled with a number, from 1 to 68; (ii) inset upper right corner – locations of all metagenomes searched in this study. Locations superimposed on an ocean chlorophyll concentration map (Aqua MODIS mission, 2010 annual composite, https://oceancolor.gsfc.nasa.gov/cgi/l3 – NASA Goddard Space Flight Center, Ocean Biology Processing Group, 2014)

To further assess the environmental distribution, we queried for the presence of cobaviruses in more than 5,000 publicly available marine metagenomes, by mapping unassembled reads to cobaviral genomes. The queried metagenomes covered a wide range of marine environments, from coastal to open oceans, and from water column, to benthic, sediment and animal associated samples. All metagenomes from the Tara Ocean Expeditions [64] were included in the dataset, as well as the viromes from Malaspina expeditions [99], along other marine datasets available in ENA in November 2017 (see SI file S4 for a complete list of all datasets used). We found cobaviruses in bonafide viromes and in metagenomes from cellular fractions, mostly in the prokaryotic range, but also in the small protist range (Fig. 5 and SI files S5). The presence of cobaviruses in cellular fractions could be explained by i) active infections at the time of sample collection, or ii) free phage particles retained on the large pore size filters by unspecific binding to the filter membrane or cell debris. A third explanation, the integration of cobaviruses in bacterial genomes as prophages, is unlikely, because, firstly, no cobaviral genes with functional annotations indicated a temperate life style. Secondly, although in phylogenetic trees using spanin cobaviruses were placed close to prophage regions from roseobacter genomes (Fig. 4a, SI file S1 Table S6), in whole genome trees cobaviruses were distant from these prophages (SI file S1 Figure S11).

Cobaviruses were detected in the euphotic water column, mainly close to coastal areas but also in the open ocean of the Pacific, Atlantic and Indian Oceans, as well as in the North Sea, the Mediterranean, the Adriatic, the Red Sea, the Arabian Sea, the Yellow Sea, the Salish Sea and in several estuaries (see Fig. 5a for an overview, SI file S5 for the list of coordinates and https://www.google.com/maps/d/viewer?mid=1LlM8bT7MSHJIzYfUY2zneec4w0vSmXNO&ll=17.833079030938055%2C-6.043723249999857&z=2 for a detailed map). These waters span temperate to tropical regions. Hot spots for cobaviruses were in bays or estuaries, with several cobaviruses being detected in these locations, for example the Goseong Bay, Delaware Estuary and Chesapeake Bay (Fig. 5b and Table 3). This is consistent with a 16 S rRNA based survey, which retrieved known cobaviral hosts mainly from coastal areas (SI file S1 Figure. S12 and Tables S4-10). Generally, abundance of cobaviruses was low. However, it increased markedly in the Port of Los Angeles samples (Fig. 5b, SI file S5), where roseophage SIO1, its related phages, and their respective host have been isolated [39].

**Table 3:** Seasonal occurrence of cobaviruses in different locations

Location		Phage	Year	Date (day.month)	Bloom situation
Helgoland		EnvZ	2010	11.05	post bloom
				18.05	post bloom
			2011	28.04	in between blooms
			2012	05.04	post bloom
				24.05	post bloom
				31.05	post bloom
				07.06	post bloom
		Env9	2012	08.03	pre bloom
			2014	20.06	n.d.^a^
Goseong Bay		SIO1	2014	10.03	n.d.
				06.12	n.d.
		VB2	2014	10.03	n.d.
		EnvX	2014	10.03	n.d.
				06.12	n.d.
		EnvY	2014	10.03	n.d.
				06.12	n.d.
		EnvZ	2014	10.03	n.d.
				06.12	n.d.
		Env9	2014	10.03	n.d.
				08.09	n.d.
				20.09	n.d.
				06.12	n.d.
		Env14	2014	10.03	n.d.
Delaware Estuary	station 36	EnvZ	2015	11.04	n.d.
	station 37	EnvZ	2015	13.04	n.d.
		Env8			
	station 38	EnvX	2015	11.04	n.d.
		EnvY			
		EnvZ			
		Env9			
	station 39	EnvX	2015	15.04	n.d.
		EnvY			
		EnvZ			
		Env9			
	station 40	EnvZ	2015	17.08	n.d.
Chesapeake Bay	station 33	EnvZ	2014	01.11	n.d.
				03.11	n.d.
		Env9	2014	22.03	n.d.
				spring	n.d.
				03.11	n.d.
	station 34	EnvZ	2014	30.08	n.d.
	station 35	EnvZ	2014	22.03	n.d.
				02.11	n.d.
				03.11	n.d.
		Env9	2014	22.03	n.d.
				03.11	n.d.

^a^n.d. = not determined

Specific cobavirus strains are cosmopolitan, as revealed by the finding of specific genomes across distant geographical locations. For example, the ICBM1 and ICBM2 phages have been isolated from the North Sea, but similar phages have been found by read mapping in metagenomes from the Australian Coast (ICBM1), and from the Goseong Bay, Yellow Sea and the Port of Los Angeles (ICBM2, Figs. 5a, b). Similarly, environmental cobaviruses have been found by read mapping not only in the metagenomes from which they were originally assembled (Table 2 and Fig. 5a), but also in many other locations (Fig. 5b). The biogeographic distribution of the cobaviruses could be explained by passive transport by oceanic currents and local selection by environmental factors shaping host communities, as proposed for marine viruses by Brum et al. [4]. In addition, considering that many positive locations for cobaviruses are also harbor areas, ship ballast water could contribute to virus transport across the oceans, in line with the findings by Kim et al. [100].

A few of the metagenomes positive for cobaviruses were part of sampling time series, allowing us to catch a glimpse of the cobaviral seasonality (see Table 3). In the North Sea, in metagenomic samples focused on spring/early summer algal blooms [16], EnvZ and Env9 were present in successive years, mostly post-bloom, but also before and during the blooms. In Goseong Bay [98] and Delaware Estuary, cobaviruses where present in early spring, late summer, fall and winter. This suggests that cobaviruses persist throughout the years in coastal environments.

### Protists as a habitat for the cobaviral hosts

We used the search for cobaviruses in microbial metagenomes (see section above), as well as glutaredoxin and RNR trees, to find indications regarding the habitat of the cobaviral hosts. Cobavirus genomes were present in several metagenomes from the protist size fractions (Fig. 5b and SI file S5), suggesting that cobaviruses infect protist-associated bacteria. Most often cobaviruses were present in the 0.8–5 µm fraction, which could arguably be contaminated with free living bacteria, but also in the >3 µm fraction (SI file S5), which makes it more likely that the bacterial cells present there were attached to or consumed by protists. The small protist size fraction is dominated by *Alveolata*, including dinoflagellates, followed by *Rhizaria* and *Stramenopila* [101], thus consisting of phagotrophic, parasitic and phototrophic species. Previous research [67] proposed that class II RNR-containing phages are infecting vitamin B12-producing bacteria associated with phototrophic protists. This was based on the phylogenetic positioning of phage class II RNRs, including that of SIO1, next to chloroviruses (viruses of the single cell green alga *Chlorella*) and microalgae, and on the cobalamin requirement by the RNR. Other studies showed that marine *Rhodobacteraceae* can be associated with protists [102–105], including close relatives of the cobaviral hosts (SI file S1 SI Tables S7 and S8).

In our own analysis, the phylogenetic neighborhood of the cobaviral glutaredoxin and RNR (Figs. 4b–e) points toward a relationship of the cobaviral hosts not only with phototrophic protists, but also with phagotrophic/mixotrophic protists, as detailed further. Interactions with phagotrophs/mixotrophs, especially amoeba, but also paramecium and dinoflagellates, are a recurring theme in the RNR and glutaredoxin trees (Figs. 4b–e). For example, several organisms found in the vicinity of cobaviruses in both glutaredoxin and RNR trees are resistant to amoeba [106–109] and, most significantly, the *Chlamydiae* are well known endosymbionts or lytic parasites of amoebae [110]. Even the chloroviruses point towards amoeba or paramecium interactions, because they infect only *Chlorella* strains that form endosymbioses with amoebae or paramecium [111, 112]. Amoebae themselves have a functional cobalamin-dependent RNR [113] (Fig. 4c) and therefore, they need partners such as the cobaviral hosts, able to synthesize vitamin B12. Many dinoflagellates are mixotrophic or heterotrophic, being able to ingest diverse prey, including bacteria [114], and their dependence on external sources of vitamin B12 has been documented previously [115–117].

Taking all this into consideration, we propose that at least some of the cobaviral hosts are frequently interacting with phagotrophic/mixotrophic protists, beyond just being grazed upon. It is possible that the cobaviral hosts, associated or not with phototrophic algae, have developed mechanisms to escape digestion in food vacuoles of predatory protists, in a similar way to amoebae-resistant bacteria [106, 107, 109, 118–120].

In their interactions with phototrophic and mixotrophic protists, marine *Rhodobacteraceae* form both mutualistic and pathogenic relationships, the latter resulting in protist lysis [121, 122]. Therefore, by exerting control on their host populations, cobaviruses could have roles in biogeochemical cycling that go beyond the release of bacterial cellular components. They could indirectly affect both marine phytoplankton growth, and thus carbon fixation, and its lysis, and thus release of the fixed organic matter in the environment. Future studies are necessary to understand the roles that cobaviruses play in the environment and their impact on roseobacter populations.

## Conclusions

This study significantly extends our knowledge of phages infecting organisms of the *Roseobacter* group, a key player in the cycling of organic matter in marine ecosystems. Using an approach that combines phage isolation with database mining for environmental phage genomes we have delineated the new Cobavirus group. Our biogeography survey included marine metagenomes from the viral, prokaryotic and protist fractions and is to date one of the largest surveys applied for a specific phage group. Cobaviruses impact *Roseobacter* populations at a global scale, from temperate to tropical marine waters, especially in coastal areas, and thus could have an influence on the biogeochemical cycling in these environments.

## Supplementary information

SI_file_S1

SI_file_S2

SI_file_S3

SI_file_S4

SI_file_S5

SI_file_S6

SI_file_S7a

SI_file_S7b

SI_file_S7c

SI_file_S7d
