# Supplementary material for: Cobaviruses – a new globally distributed phage group infecting Rhodobacteraceae in marine ecosystems
Source: ISME J. 2019 Feb 4;13(6):1404–21. doi: 10.1038/s41396-019-0362-7 (PMC6775973; doi:10.1038/s41396-019-0362-7)
Supplement: Supplementary file 1 — SI_file_S1 [file 41396_2019_362_MOESM1_ESM.pdf]

# Cobaviruses – a new globally distributed phage group infecting *Rhodobacteraceae* in marine ecosystems

Vera Bischoff[1], Boyke Bunk[2], Jan P. Meier-Kolthoff[2], Cathrin Spröer[2], Anja Poehlein[3], Marco Dogs[1], Mary Nguyen[4], Jörn Petersen[2], Rolf Daniel[3], Jörg Overmann[2], Markus Göker[2], Meinhard Simon[1], Thorsten Brinkhoff[1] and Cristina Moraru[1*]

[1] Institute for Chemistry and Biology of the Marine Environment, Carl-von-Ossietzky –Str. 9 -11, D-26111 Oldenburg

[2] Leibniz-Institut DSMZ – Deutsche Sammlung von Mikroorganismen und Zellkulturen GmbH, Inhoffenstraße 7 B, 38124 Braunschweig

[3] Georg-August-University Goettingen, Institute of Microbiology and Genetics, Department of Genomic and Applied Microbiology & Goettingen Genomics Laboratory, Grisebachstr. 8, D-37077 Goettingen

[4] Centre for Marine Bio-Innovation, School of Biological, Earth and Environmental Sciences, The University of New South Wales, Kensington, NSW, Australia

*corresponding author:

Cristina Moraru, email: liliana.cristina.moraru@uni-oldenburg.de, telephone: 0049 441 798 3416 Fax +49-(0)441-798-3438

# Supplementary information

# Experimental procedures

## *Cultivation media.*

Liquid cultures of the bacterial host, *Lentibacter* sp. SH36 ([1]), were grown in artificial saltwater medium (1x ASW) (24.32 g/l NaCl, 10 g/l $MgCl_2x6H_2O$, 1.5 g/l $CaCl_2x2H_2O$, 0.66 g/l KCl, 4 g/l $Na_2SO_4$, 2.38 g/l HEPES, 0.6 g/l peptone, 0.3 g/l yeast extract, 84 mM KBr, 40 mM $H_3BO_3$, 15 mM $SrCl_2$, 40 mM $NH_4Cl$, 4 mM $KH_2PO_4$, 7 mM NaF, pH 7.5), which was autoclaved and completed before use with 1 ml/l of sterile filtered multi vitamin solution (after (Balch et al. 1979)), 0.25 ml/l of sterile filtered trace element solution A (1.5 g $FeCl_2x4H_2O$ in 10 ml 25 % HCl and 250 ml MilliQ water) and 0.1 ml/l of autoclaved trace element solution B (19 mg/l $CoCl_2x6H_2O$, 10 mg/l $MnCl_2x2H_2O$, 7 mg/l $ZnCl_2$, 3.6 mg/l $Na_2MoO_4x2H_2O$, 2.4 mg/l $NiCl_2x6H_2O$, 0.6 mg/l $H_3BO_3$, 0.2 mg/l $CuCl_2x2H_2O$).

The solid media used for plaque assays was Marine Broth. This media had the following recipe. 5.0 g/l peptone, 1.0 g/l yeast extract, 0.1 g/l $C_6H_8FeO_7$, 12.6 g/l $MgCl_2x6H_2O$, 3.24 g/l $Na_2SO_4$, 19.45 g/l NaCl, 2.38 g/l $CaCl_2x2H_2O$, 0.55 g/l KCl, 0.16 g/l $NaHCO_3$, 0.01 g/l $Na_2HPO_4x2H_2O$, 0.008 g/l KBr, 0.034 g/l $SrCl_2x6H_2O$, 0.022 g/l $H_3BO_3$, 0.007 g/l $Na_2SiO_3x3H_2O$, 0.0024 g/l NaF, 0.0016 g/l $NH_4NO_3$.

## *Purification of ICBM1 and ICBM2 phages.*

Bacterial cells and their debris from the phage enrichments were removed by a centrifugation step (15 min, 4000 x g, 20 °C), followed by 0.2 µm filtration (0.2 µm, Rotilabo-syringe filters, Carl Roth) of the supernatant. The ICBM1 and ICBM2 phages were isolated from the enriched phage fractions by obtaining single phage plaques.

To obtain single plaques, serial dilutions ($10^0$, $10^{-1}$, etc.) were prepared from the phage fractions by mixing with ASW base (ASW media without yeast extract, peptone and vitamins). 100 µl of phage dilution were mixed with 280 µl of exponentially growing host culture (OD = 0.2-0.3) and incubated for 15 min on ice. Afterwards, the mixture was transferred to 3 ml MB-soft agar (0.6 % low melting point Biozym Plaque GeneticPure agarose, Biozym, kept warm at 37°C), mixed by brief vortexing and poured onto the bottom MB agar layer (1.8 % agar). After drying of the top layer, the plates were incubated for three days at 20 °C. For isolation, when phage plaques were observed as clearing zones within the grown bacterial lawn, they were picked with sterile Pasteur pipettes and incubated overnight in 500 µl ASW base at 4 °C. After subsequent centrifugation (10 min, 10000 x g, 4 °C), the supernatant was used for a next round of plaque assays. This procedure of plaque assay, picking of plaques and re-plating was repeated three times to ensure purity of the newly isolated phages.

The ICBM1 and ICBM2 phages were stored either as phage lysate at +4°C or as glycerol stock of free phages or infected cells at -80°C (for details, see below).

## Spot assays.

To estimate phage numbers, serial dilutions ($10^0$, $10^{-1}$, etc.) were prepared from the phage fractions by mixing with ASW base (ASW media without yeast extract, peptone and vitamins). 280 µl of exponentially growing host culture (OD = 0.2-0.3) were mixed with 3 ml MB-soft agar (kept warm at 37°C) and poured onto the bottom MB agar layer. After drying of the top layer, 10 µl of each phage fraction dilution were pipetted on top as droplets. The plates were incubated at 20 °C.

## *Preparation of phage ICBM1 and ICBM2 stocks.*

In two Erlenmeyer flasks, 20 ml 1xASW medium was inoculated with an exponentially growing *Lentibacter* sp. SH36 culture (final OD = 0.006). One culture was infected with 300 µl of the phage ICBM1 supernatant, the other was not infected and regarded as control. Both cultures were incubated at 20 °C and 100 rpm overnight, until bacterial lysis in the infected culture was indicated by low $OD_{600}$

(compared with the control culture) and disrupted cell particles. The phage fraction was obtained by removing cells and debris by centrifugation (15 min, 4000 x g, 20 °C), followed by 0.22 µm filtration of the supernatant. The phage fraction was stored at +4°C. For long term storage, two types of glycerol stocks were prepared: i) stock of free phage particles (1 part phage fraction and 1 part MB media with 50% glycerol) and ii) stock of infected host cells (1 part infected cells - 375 µl phage fraction added to 375 µl host culture, 15 min on ice for absorption - and 1 part MB media with 50% glycerol).

### *TEM.*

Phages were concentrated from the ICBM1 or ICBM2 phage lysate by polyethylene glycol (PEG) precipitation. 150 ml phage fraction resulting from two subsequent infections (see above) were incubated for 2 h at 4 °C with PEG (final concentration 10%) and NaCl (final concentration 0.6 mM). After centrifugation for 2 h at 7197 x g and 4 °C, the supernatant was discarded and the pellet resuspended in 500 µl SM buffer (100 mM NaCl, 8 mM MgSO4, 50 mM Tris-HCl pH 7.4) in total. For resuspension of the phages, 30 min incubation at 4 °C followed. PEG was removed by mixing with an equal volume of 100 % chloroform, shaking for 5 min and incubation on ice for 5 min. After centrifugation (10 min, 3000 x g, 4 °C), the upper layer was collected.

Further concentration and purification of the phages was done by cesium chloride gradient ultracentrifugation. In UltraClear™ centrifuge tubes, a density gradient was set up from cesium chloride solutions with different densities (from bottom up): 1.5 ml of 1.65 g/ml, 2 ml of 1.5 g/ml, 2 ml of 1.4 g/ml, 1 ml of 1.2 g/ml. The PEG concentrated phage fraction was transferred on top. Ultracentrifugation was run for 4 h at 20 °C and 25000 rpm (Beckman, SW 41 Ti). Afterwards, the visible band corresponding to the phages was collected with syringe and needle through the side wall of the ultracentrifuge tube (~500 µl). Removal of cesium chloride was done by dialysis with Slide-A-Lyzer® G2 Dialysis Cassettes 10K MWCO (ThermoScientific) against ASW base for 21 h with buffer exchange after 3 h and 18 h.

30 µl of phage ICBM1 or ICBM2 concentrate were pipetted on top of a carbon coated grid (Formvar 162, 200 mesh) and phages were allowed to absorb for 3 min, followed by staining with 30 µl uranylacetate (2 %) or ammonium molybdate (2 %) for 45 sec and gentle removal of the liquid with filter paper. After air drying for 15 min, the grids were visualized with the transmission electron microscope Zeiss EM902A. Images were documented with the Proscan High Speed SSCCD camera and analyzed using the software ImageSP viewer (Version 1.2.5.16).

### *DNA extraction from the ICBM1 phage.*

Phages were concentrated from the phage lysates by precipitation with polyethylene glycol. For this purpose, 4 x 25 ml phage lysate prepared as above (with one exception: to avoid phage loss, cells and debris were removed only by centrifugation, and not by filtration) were mixed with 50% PEG (final concentration 10 %) and 5M sodium chloride (final concentration 0.6 M) and incubated for 2 h at 4 °C. After centrifugation (2 h, 7197 x g, 10 °C) the phage pellets were resuspended in 500 µl SM buffer (100 mM NaCl, 8 mM MgSO4, 50 mM Tris-HCl pH 7.4) each. Extracellular DNA was removed by incubating the phage concentrates with 0.04 units/µl of Turbo DNase (Invitrogen, Ambion) for 30 min at 37°C, followed by enzyme inactivation by incubating for 10 min at 75 °C with 15 mM EDTA. Further, the phage DNA was extracted using the ChargeSwitch gDNA Mini Bacteria Kit (ThermoFisher Scientific), according to the instructions manual, including the with the RNase digestion, but with the exception of no lysosyme in the first step. The DNA was finally eluted in 1 ml elution buffer. The concentration and quality of the obtained DNA was checked fluorometrically with Qubit 2.0 and the Qubit® dsDNA

HS Assay, spectrophotometrically with Nanodrop 2000 spectrophotometer and visually by regular gel electrophoresis (0.7 % agarose gel, 50 V, SYBR Gold staining).

### *DNA extraction of the intracellular phage fraction from the phage enrichments.*

To extract the intracellular phage fraction from the phage enrichment, when lysis was observed, the cells from the enrichments and the positive control were retrieved by centrifugation (15 min, 4000 x g, 20 °C). The cell pellets were embedded in agarose plugs by mixing with SeaKem Gold Agarose for PFGE, Lonza Rockland Inc. (final concentration 0.8 %), distributing the mixture into 100 µl molds and allowing it to solidify for 30 min at 4 °C. Plugs were collected in a 50 ml Falcon tube and incubated overnight at 50 °C in 2 ml ESP buffer (1 % N-laurylsarcosine, 1 mg/ml proteinase K, 0.5 M EDTA pH 9.0). Afterwards, the ESP was discarded, the plugs were washed three times with TE buffer (10 mM Tris-HCl pH 8.0, 2 mM EDTA pH 9.0) and stored in TE buffer at 4 °C until further use. The DNA from the agarose plugs was separated during agarose gel electrophoresis (1 % SeaPlaque GTG Agarose, Lonza Rockland Inc., TAE buffer - 40 mM Tris-acetate, 1 mM EDTA, pH 8.3, migration 2 h at 60 V). Afterwards, the gel was cut into two halves. One half was stained with Ethidium bromide bath (1 µg/ml, 30 min) and documented with BioDocAnalyze system (Biometra). The distance from the loading pocket to the phage DNA band was measured and used to localize the phage DNA band in the unstained half and to cut it out from the gel. The agarose pieces were stored overnight in TE buffer (10 mM Tris-HCl pH 8.0, 5 mM EDTA pH 9.0), followed by agarose digestion with 1 unit of β-agarase (New England Biolabs Inc.), according to the manufacturer's protocol. Undigested agarose was removed by centrifugation for 10 min at 20,000 x g. The obtained supernatants were concentrated to 100 µl each using 100 kDa Amicon Ultra centrifugal filters (0.5 ml volume, Merck Millipore) and then stored at -20 °C. Afterwards, an additional purification step was performed using the ChargeSwitch gDNA Mini Bacteria Kit (ThermoFisher Scientific) for DNA extraction. This was done according to the kit manual (including the RNase digestion, but without lysozyme) and the DNA was finally eluted in 100 µl elution buffer. Concentration and quality of the obtained DNA were checked with Qubit 2.0 fluorometer, Nanodrop 2000 spectrophotometer and by regular gel electrophoresis (0.7 % agarose gel, 50 V, Ethidium bromide staining).

### *Illumina genome sequencing.*

The extracted DNA from the ICBM1 and ICBM2 phages and the phage enrichments was used to generate Illumina NexteraXT shotgun paired-end sequencing libraries, which were sequenced with a MiSeq instrument and the MiSeq reagent kit version 3, as recommended by the manufacturer (Illumina, San Diego, CA, USA). For quality-filtering, Trimmomatic version 0.35 (Bolger et al. 2014) or Bbduk from the BBTools package (BBTools (https://jgi.doe.gov/data-and-tools/bbtools/) were used. The assembly was performed with the SPAdes genome assembler software version 3.9.0 (Bankevich et al. 2012) and the read coverage of the whole assembly determined with QualiMap version 2.1 (García-Alcalde et al. 2012). In addition, the read mapping of enrichments reads on individual phage genomes was done BBMap from BBTools package.

### *PacBio library preparation, sequencing and assembly.*

SMRTbell template library was prepared according to the instructions from Pacific Biosciences, Menlo Park, CA, USA, following the Procedure & Checklist Greater than 10 kb Template Preparation and Sequencing. Briefly, for preparation of 10kb libraries ~4µg of each phage DNA was sheared using a Covaris S220, Woburn, MA, USA according to the manufacturer´s instructions. DNA was end-repaired and ligated overnight to barcoded SMRTbell adapters applying components from the DNA/Polymerase

Binding Kit P6 from Pacific Biosciences (Menlo Park, CA, USA). Reactions were carried out according to the instructions of the manufacturer. One part VB-1 SMRTbell template was combined with 2.5 parts Ex53-3 SMRTbell template. BluePippin Size-Selection to greater than 4 kb was performed according to the manufacturer´s instructions (Sage Science, Beverly, MA, USA). Conditions for annealing of sequencing primers and binding of polymerase to purified SMRTbell template were assessed with the Calculator in RS Remote, PacificBiosciences, Menlo Park, CA, USA. SMRT sequencing was carried out on the PacBio *RSII* (PacificBiosciences, Menlo Park, CA, USA) taking one 240-minutes movie for one SMRT cell. Long read genome assemblies of all three phages have been performed using the HGAP4 Whitelisting protocol (https://github.com/PacificBiosciences/Bioinformatics-Training/wiki/HGAP-Whitelisting-Tutorial) within SMRTPipe 2.3.0 applying a genome size of 100kb and a minimum subread length of 1kb after demultiplexing using the RS_Subreads.1 protocol contained within SMRT Portal 2.3.0.

## Results

### Retrieval of similar phage genomes from public sequence datasets.

To find cultured and environmental relatives of the three phages, the following datasets were queried for related sequences: i) the Tara Ocean Viromes (Brum et al. 2015), ii) the Earth Virome (Paez-Espino et al. 2016), iii) the Global Ocean Virome (GOV, (Roux et al. 2016)), iv) the IMG/VR (Paez-Espino et al. 2017) and v) the Environmental Viral Genomes (Nishimura et al. 2017). Six highly similar genomes belonged to cultivated phages: the P12053L phage (38889 bp) infecting *Celeribacter marinus* IMCC12053$^T$, the SIO1 phage (39 898 bp) infecting *Roseobacter* sp. SIO67 and four other SIO1 related strains, infecting *Roseobacter* sp. SIO67 and *Roseobacter* sp. GAI-101 (Angly *et al.* 2009). The last four phages had incomplete genomes, with several regions of uncertainty (long N stretches). Therefore, they were included in the phylogenetic analysis as draft genomes, but excluded from further genomic analysis. Several other genomes represented environmental fragments of different lengths. Only complete or close to complete genomes (>35 kbps, no long N stretches) were considered for further phylogenetic and genomic analyses. One of these circularized due to terminal redundancies, indicating genome completeness - EnvX (40752 bp), and five of them were incomplete, but close in size to the complete genomes and contiguous - Env9 (41607 bp), EnvY (36003 bp), EnvZ (35824 bp), Env8 (38447 bp) and Env14 (35006 bp). EnvX, EnvY, EnvZ, Env8 and Env9 were retrieved from IMG/VR /Earth Virome datasets. Env14 was retrieved from the GOV dataset (see Table 2).

### Genome termini and genome rearrangements..

**ICBM1, ICBM2 and ICBM3.** We sequenced the three phages by Illumina and PacBio, as isolates or from the enrichments (Table S2). The three phage genomes were assembled as circular contigs of ~ 40kbps. Sequencing coverage data was used to determine the ends of the genomes. With Illumina sequencing, a sharp drop in coverage was noticed for ICBM1 and ICBM2 genomes, which could indicate linear, non-circularly permuted genomes, with cohesive ends (Merrill et al. 2016), (Figure S3). On the other hand, for both genomes, we noticed a region of low coverage and high %GC (see Figure S3). The low coverage could be due to the library preparation with the NexteraXT kit, which is known to have low performance at the genome ends (Illumina 2015). Furthermore, the high %GC could contribute to the drop in coverage (Aird et al. 2011). To elucidate the genome ends, we turned to PacBio single molecule real time (SMRT) sequencing, because native DNA is used and thus, no PCR bias is observed. Furthermore, much longer read lengths can be retrieved, which facilitates assembly, especially important for mixed samples, for example the phage enrichments. Thus, the artificial redundancies produced by the assembler at the end of the three phage contigs were larger, having a size of ~5kb, which is equal to the mean subread length achieved with our PacBio sequencing approach (Figure S4). In all three phage genomes, short DTRs of about 160 bps were easily recognized as spikes in coverage (Figure S4 A). To delineate the final genome structure of the three phages, artificial redundancies were removed and the phage genome was adjusted to their direct terminal repeats (Figure S4 C). The exact genome start and stop positions were derived from long read mappings by a detailed inspection of the respective regions in Integrative Genome Viewer (Robinson et al. 2011) (Figure S5). The sharp drop in coverage in the Illumina assemblies corresponded to a GC rich region of the DTRs (see Figure S3), and thus, explained the apparent discrepancy between the Illumina and PacBio read coverage data.

We used the DTRs from ICBM1 to define the genome termini and rearrange the gene order accordingly, not only for the SIO1 phage, but also for the P12053L and environmental cobaviruses.

**SIO1.** A search with the ICBM1 DTR in the SIO1 genome revealed the presence of a similar region (80% nucleotide identity) at position 8716-8891. Based on this approach, base 8716 from the original

SIO1 genome became base 1 in the reordered genome, with the left side being concatenated at the end of the right side, and the ICBM1 phage DTR homologous region being added also at the right end, as depicted in Fig. S5. Similar regions were found in all four SIO1-related phages isolated by (Angly et al. 2009), having 90% to 99% nucleotide identity with the SIO1 DTR.

**P12053L.** A search with the ICBM1 DTR in the P12053L genome found a similar region (~94% identity) at position 244-415. Therefore, the genome was rearranged in a similar way to SIO1, with base 244 becoming base 1. No gene rearrangement was necessary in this case. The DTR sequence was determined by homology to ICBM1 DTR and added at the right end of the genome also.

**Environmental cobaviruses.** For two of the environmental genomes (EnvX and Env9) we determined the genome start by finding regions with high identity with the left region of the ICBM2 DTR (>70% over 49 nt, see Figure S9). However, the complete sequence of the DTRs could not be established, because of the low similarity over the remaining alignment (~50% identity). Env9 was not circular, but because we established the start at position 3404, we plotted the position 1-3403 at the end of the genome in Figure 3. The remaining environmental genomes showed no regions of similarity with the ICBM1 or ICBM2 phage DTRs, presumably due to their incompleteness. The phylogenetic positioning both in the GBPD and terminate trees strongly support the presence of DTRs at as genome termini for all the environmental genome.

**Table S1: List of *Rhodobacteraceae* strains used for the host range assay.**

| Name | Strain designation | Strain | Infected (- no, + yes) |
|---|---|---|---|
| *Antarctobacter heliothermus* | EL-219 | DSM 11445 | - |
| *Celeribacter baekdonensis* | L-6 | DSM 27375 | - |
| *Celeribacter indicus* | P73 | DSM 27257 | - |
| *Celeribacter marinus* | IMCC12053 | DSM 100036 | - |
| *Celeribacter neptunius* | H 14 | DSM 26471 | - |
| *Citreicella aestuarii* | AD8 | DSM 22011 | - |
| *Citreicella marina* | CK-I3-6 | DSM 26424 | - |
| *Dinoroseobacter shibae* | 5 Plasmids | DSM 16493 | - |
| *Huaishuia halophila* | ZXM137 | DSM 26270 | - |
| *Hwanghaeicola aestuarii* | Y26 | DSM 22009 | - |
| *Jannaschia donghaensis* | DSW-17 | DSM 102233 | - |
| *Jannaschia helgolandensis* | Hel10 | DSM 14858 | - |
| *Jannaschia pohangensis* | H1-M8 | DSM 19073 | - |
| *Jannaschia rubra* | 4SM3 | DSM 16279 | - |
| *Leisingera aquimarina* | R-26159 | DSM 24565 | - |
| *Leisingera caerulea* | 13 | DSM 24564 | - |
| *Leisingera daeponensis* | TF-218 | DSM 23529 | - |
| *Leisingera methylohalidivorans* | MB2 | DSM 14336 | - |
| *Lentibacter sp.* | SH36 | | |
| *Litoreibacter albidus* | KMM 3851 | DSM 26922 | - |
| *Litoreibacter arenae* | GA2-M15 | DSM 19593 | - |
| *Litoreibacter janthinus* | KMM 3842 | DSM 26921 | - |
| *Litorimicrobium taeanense* | G4 | DSM 22007 | - |
| *Loktanella cinnabarina* | LL-001 | DSM 29954 | - |
| *Loktanella fryxellensis* | R-7670 | DSM 16213 | - |
| *Loktanella hongkongensis* | UST950701-009P | DSM 17492 | - |
| *Loktanella koreensis* | GA2-M3 | DSM 17925 | - |
| *Loktanella pyoseonensis* | JJM85 | DSM 21424 | - |
| *Loktanella salsilacus* | R-8904 | DSM 16199 | - |
| *Loktanella tamlensis* | SSW-35 | DSM 26879 | - |
| *Loktanella vestfoldensis* | R-9477 | DSM 16212 | - |
| *Maribius pelagius* | B5-6 | DSM 26893 | - |
| *Maribius salinus* | CL-SP27 | DSM 26892 | - |
| *Marinovum algicola* | FF3 | DSM 10251 | - |
| *Marinovum algicola* | DG898 | DSM 27768 | - |
| *Maritimibacter alkaliphilus* | HTCC2654 | DSM 100037 | - |
| *Nautella italica* | R11 | DSM 26436 | - |
| *Oceanibulbus indolifex* | HEL-45 | DSM 14862 | - |
| *Oceanicola batsensis* | HTCC2597 | DSM 15984 | - |
| *Oceanicola granulosus* | HTCC2516 | DSM 15982 | - |
| *Oceanicola nanhaiensis* | SS011B1-20 | DSM 18065 | - |
| *Octadecabacter temperatus* | SB1 | DSM 26878 | - |
| *Palleronia marisminoris* | B33 | DSM 26347 | - |
| *Pelagibaca bermudensis* | HTCC2601 | DSM 26914 | - |
| *Phaeobacter gallaeciensis* | BS 107 | DSM 26640 | - |
| *Phaeobacter inhibens* | | DSM 17395 | - |

| | | | |
|---|---|---|---|
| *Phaeobacter inhibens* | T5 | DSM 16374 | - |
| *Phaeobacter inhibens* | 2.10 | DSM 24588 | - |
| *Ponticoccus litoralis* | CL-GR66 | DSM 18986 | - |
| *Pseudophaeobacter arcticus* | 20188 | DSM 23566 | - |
| *Pseudoruegeria lutimaris* | HD-43 | DSM 25294 | - |
| *Roseibacterium elongatum* | Och 323 | DSM 16469 | - |
| *Roseivivax isoporae* | sw2 | DSM 22223 | - |
| *Roseivivax roseus* | BH87090 | DSM 23042 | - |
| *Roseobacter denitrificans* | Och 114 | DSM 7001 | - |
| *Roseobacter litoralis* | Och 149 | DSM 6996 | - |
| *Roseovarius crassostreae* | CV919-312, CVSP | DSM 16950 | - |
| *Roseovarius halocynthiae* | MA1-10 | DSM 27840 | - |
| *Roseovarius indicus* | B108 | DSM 26383 | - |
| *Roseovarius lutimaris* | 112 | DSM 28463 | - |
| *Roseovarius marinus* | HDW-9 | DSM 25228 | - |
| *Roseovarius mucosus* | DFL-24 | DSM 17069 | - |
| *Roseovarius nubinhibens* | ISM | DSM 15170 | - |
| *Ruegeria atlantica* | 1480 | DSM 5823 | - |
| *Ruegeria conchae* | TW15 | DSM 29317 | - |
| *Ruegeria marina* | ZH17 | DSM 24837 | - |
| *Ruegeria pomeroyi* | DSS-3 | DSM 15171 | - |
| *Sagittula stellata* | EE-37 | DSM 11524 | - |
| *Salinihabitans flavidus* | ISL-46 | DSM 27842 | - |
| *Salipiger mucosus* | A3 | DSM 16094 | - |
| *Sedimentitalea nanhaiensis* | NH52F | DSM 24252 | - |
| *Sediminimonas qiaohouensis* | YIM B024 | DSM 21189 | - |
| *Shimia haliotis* | WM35 | DSM 28453 | - |
| *Shimia marina* | CL-TA03 | DSM 26895 | - |
| *Sulfitobacter delicatus* | KMM 3584 | DSM 16477 | - |
| *Sulfitobacter dubius* | KMM 3554 | DSM 16472 | - |
| *Sulfitobacter litoralis* | Iso 3 | DSM 17584 | - |
| *Sulfitobacter marinus* | SW-265 | DSM 23422 | - |
| *Sulfitobacter mediterraneus* | CH-B427 | DSM 12244 | - |
| *Sulfitobacter noctilucae* | NB-68 | DSM 100978 | - |
| *Sulfitobacter noctilucicola* | NB-77 | DSM 101015 | - |
| *Sulfitobacter pseudonitzschiae* | H3 | DSM 26824 | - |
| *Sulfitobacter sp.* | EE-36 | DSM 11700 | - |
| *Thalassobius aestuarii* | JC2049 | DSM 15283 | - |
| *Thalassobius maritimus* | GSW-M6 | DSM 28223 | - |
| *Thalassococcus halodurans* | UST050418-052 | DSM 26915 | - |
| *Thioclava dalianensis* | DLFJ1-1 | DSM 29618 | - |
| *Thioclava pacifica* | TL 2 | DSM 10166 | - |
| *Tranquillimonas alkanivorans* | A34 | DSM 19547 | - |
| *Tropicibacter multivorans* | MD5 | DSM 26470 | - |
| *Tropicibacter naphthalenivorans* | C02 | DSM 19561 | - |
| *Tropicimonas isoalkanivorans* | B51 | DSM 19548 | - |
| *Wenxinia marina* | HY34 | DSM 24838 | - |
| *Yangia pacifica* | DX5-10 | DSM 26894 | - |

**Table S2: Sequencing and assembling phage genomes from isolates and enrichments**

| | Phage isolates – sequenced by | | Enrichments – sequenced by | |
|---|---|---|---|---|
| | **Illumina** | **PacBio** | **Illumina (S1 and S2)** | **PacBio (S2 only)** |
| **ICBM1** | yes | yes | not assembled | not assembled |
| **ICBM2** | yes | no | assembled in S2 | assembled in S2 |
| **ICBM3** | n.a. | n.a. | assembled in S1, S2 | assembled in S2 |

**Table S3: Cobaviral genomes - % GC and length (bases).**

| Name | %GC | Sequence Length (bases) |
|---|---|---|
| vB_LenP_ICBM1 | 47.00% | 40163 |
| vB_LenP_ICBM2 | 47.80% | 40907 |
| vB_LenP_ICBM3 | 47.30% | 40498 |
| SIO1 | 46.20% | 40072 |
| P12053L | 46.10% | 39061 |
| EnvX | 40.20% | 40752 |
| EnvY | 40.10% | 36003 |
| EnvZ | 44.80% | 35824 |
| Env8 | 39.80% | 38447 |
| Env9 | 40.30% | 41607 |
| Env14 | 40.30% | 35066 |

**Table S4: Bi-directional rho-independent transcriptional terminators in the genomes of the Cobavirus group (blue - stems, red - loops).**

| phage | Terminat or name | Position | sequence | strand | delta G |
|---|---|---|---|---|---|
| ICBM1 | t1 | 15927-15979 | CAAATAAGTAAAGCCCCCAAGGAGAAATCCAAGGGGGCTaTTTCTTTGTGTAT | - | -13.84 |
| ICBM1 | t2 | 15930-15981 | CACAAAGAAATAGCCCCCTTGGATTTCTCCTTGGGGGCTTTACTTATTTGGA | + | -13.64 |
| ICBM3 | t1 | 15916-15968 | CAAATAAGTAAAGCCCCCAAGGAGAAATCCAAGGGGGCTaTTTCTTTATGTAT | - | -13.84 |
| ICBM3 | t2 | 15919-15970 | CATAAAGAAATAGCCCCCTTGGATTTCTCCTTGGGGGCTTTACTTATTTGGA | + | -13.64 |
| ICBM2 | t1 | 14733-14782 | AACACAAAGAAGCCCCCAAGGAGAAATCCAAGGGGGCTTTTGCTTGTCTA | - | -12.64 |
| ICBM2 | t2 | 14735-14784 | GACAAGCAAAAGCCCCCTTGGATTTCTCCTTGGGGGCTTCTTTGTGTTTA | + | -13.14 |
| P12053L | t1 | 17261-17316 | TAAACACAAAGAAGCCCCCAAGGATTTTACTCCAAGGGGGCTTTTGCTTGTTCATC | - | -14.09 |
| P12053L | t2 | 17265-17316 | AACAAGCAAAAGCCCCCTTGGAGTAAAATCCTTGGGGGCTTCTTTGTGTTTA | + | -12.99 |
| SIO1 | t1 | 15149-15204 | TAAACACAAAGAAGCCCCCAAGGATTAATCTCCAAGGGGGCTTTTGTTTGTCTATA | - | -14.09 |

| | | | | | |
|---|---|---|---|---|---|
| SIO1 | t2 | 15153-15204 | GACAAACAAAAGCCCCCTTGGAGATTAATCCTT GGGGGCTTCTTTGTGTTTA | + | -12.99 |
| EnvX | t1 | 12131-12185 | GAAATAAAGAAGAAGCCCCAAGGAGAAATCCT GAGGGGCTTTTTTATTACTCTTG | - | 12.06 |
| EnvX | t2 | 12134-12187 | GAGTAATAAAAAAGCCCCTCAGGATTTCTCCTT GGGGCTTcTTCTTTATTTCTT | + | -11.26 |
| EnvY | t1 | 12048-12102 | GAAATAAAGAAGAAGCCCCAAGGAGAAATCCT GAGGGGCTTTTTTATTACTCTTG | - | -12.06 |
| EnvY | t2 | 12051-12104 | GAGTAATAAAAAAGCCCCTCAGGATTTCTCCTT GGGGCTTcTTCTTTATTTCTT | + | -11.26 |
| Env8 | t1 | 11722-11764 | AGAAAAAGTAAGGGAGCCTAAGTAGCTCCCcTT TTTTATACCT | - | -10.60 |
| Env8 | t2 | 11724-11765 | GTATAAAAAAGGGGAGCTACTTAGGCTCCCTTA CTTTTTCTT | + | -12.70 |
| Env9 | t1 | 18115-18164 | AAATAAAATAAACCCCCTTGGATTTCTCCTTGGG GGTTTTTTCTTACTTG | - | -10.44 |
| Env9 | t2 | 18115-18169 | CAAGTAAGAAAAAACCCCCAAGGAGAAATCCA AGGGGGTTTaTTTTATTTCTTTT | + | -12.34 |
| Env14 | t1 | 12255-12297 | TAAAAGAAGAAGGGAGCCTAAGTAGCTCCCcTT TTTTTTATGC | - | -10.60 |
| Env14 | t2 | 12257-12298 | ATAAAAAAAAGGGGAGCTACTTAGGCTCCCTTC TTCTTTTAA | + | -12.70 |

**Table S5: DTRs from related phages.**

| Group | phages | DTRs - length | DTRs - nucleotide identity (excluding gaps) | DTRs - alignment |
|---|---|---|---|---|
| 1 | Acinetobacter phage phiAB1 | 410 | 87% | 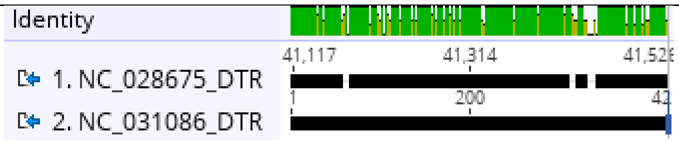 |
| | Acinetobacter phage phiAB6 | 421 | | |
| 2 | Yersinia phage Berlin | 227 | 95% | 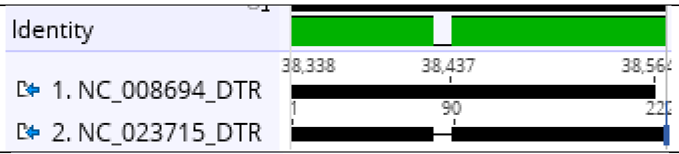 |
| | Yersinia phage Yep-phi | 222 | | |
| 3 | Pseudomonad phage gh-1 | 217 | 92% | 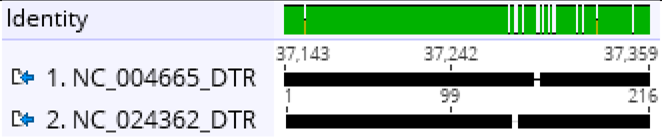 |
| | Pseudomonas phage phiPSA2 | 216 | | |
| 4 | Yersinia phage phiA1122 | 148 | 80% | 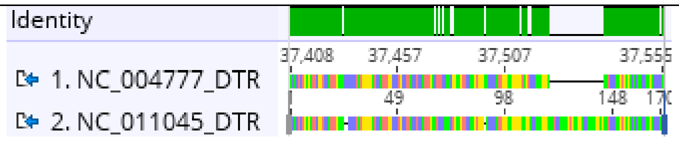 |
| | Enterobacteria phage 13a | 170 | | |
| 5 | Citrobacter phage SH5 | 191 | 100% | 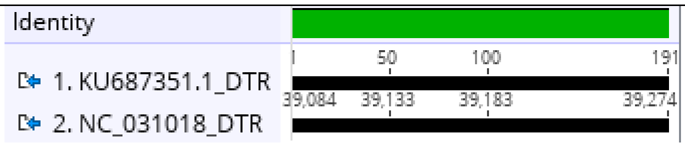 |
| | Citrobacter phage SH4 | 191 | | |
| 6 | Klebsiella phage K11 | 180 | 83% | 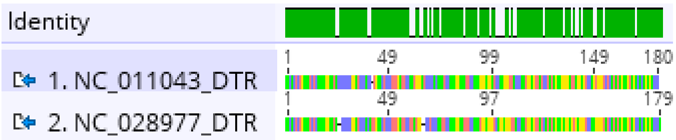 |
| | Klebsiella phage vB_KpnP_KpV289 | 179 | | |
| 7 | Enterobacteria phage K30 | 393 | 80% | 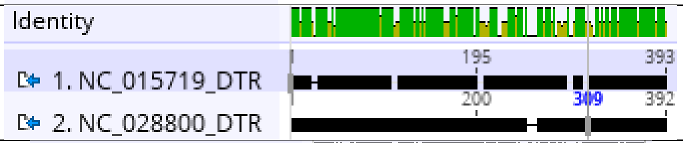 |
| | Klebsiella phage K5 | 392 | | |
| 8 | Enterobacteria phage BA14 | 194 | 83% - 93% | 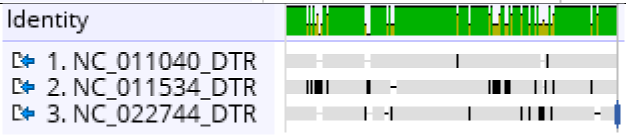 |
| | Kluyvera phage Kvp1 | 194 | | |
| | Erwinia phage FE44 | 193 | | |
| 9 | Pseudomonas phage PT5 | 413 | 98% - 100% | 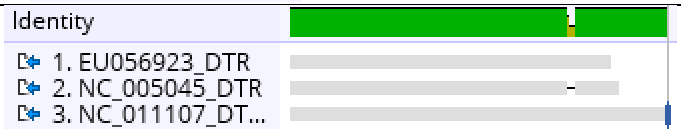 |
| | Pseudomonas phage phiKMV | 414 | | |
| | Pseudomonas phage PT2 | 488 | | |
| 10 | Klebsiella phage KP34 | 216 | 71% - 87% | 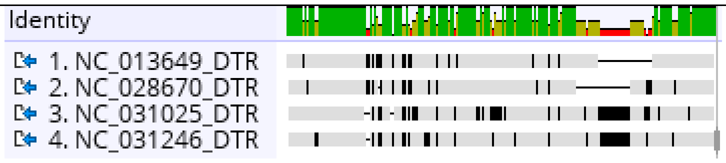 |
| | Klebsiella phage vB_KpnP_KpV41 | 214 | | |
| | Klebsiella phage vB_KpnP_KpV475 | 243 | | |
| | Klebsiella phage vB_KpnP_KpV71 | 246 | | |

<table>
<tr><td rowspan="4">11</td><td>Enterobacteria phage EcoDS1</td><td>178</td><td rowspan="4">84% - 93%</td><td rowspan="4">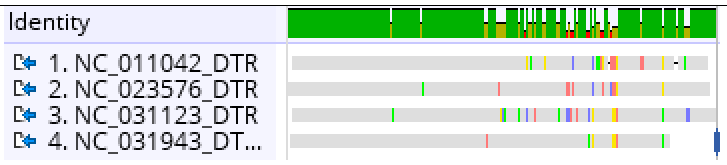
</td></tr>
<tr><td>Citrobacter phage CR44b</td><td>183</td></tr>
<tr><td>Citrobacter phage SH3</td><td>184</td></tr>
<tr><td>Escherichia phage vB_EcoP_GA2A</td><td>165</td></tr>
<tr><td rowspan="5">12</td><td>Pseudomonas phage LUZ24</td><td>184</td><td rowspan="5">88% - 99%</td><td rowspan="5">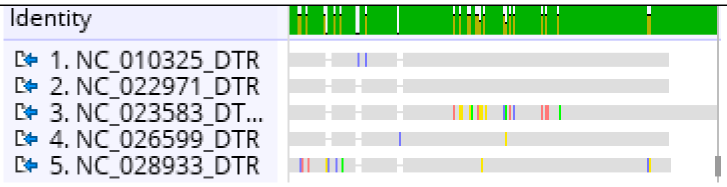
</td></tr>
<tr><td>Pseudomonas phage phiIBB-PAA2</td><td>183</td></tr>
<tr><td>Pseudomonas phage TL</td><td>207</td></tr>
<tr><td>Pseudomonas phage vB_PaeP_C2-10_Ab22</td><td>184</td></tr>
<tr><td>Pseudomonas phage PhiCHU</td><td>185</td></tr>
<tr><td rowspan="7">13</td><td>Enterobacteria phage T7M</td><td>230</td><td rowspan="7">83% - 100%</td><td rowspan="7">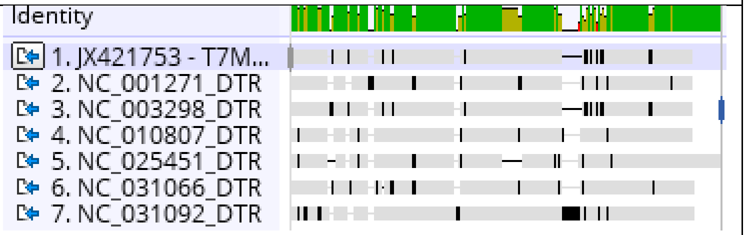
</td></tr>
<tr><td>Yersinia phage phiYeO3-12</td><td>232</td></tr>
<tr><td>Enterobacteria phage T3</td><td>231</td></tr>
<tr><td>Salmonella phage phiSG-JL2</td><td>230</td></tr>
<tr><td>Yersinia phage vB_YenP_AP5</td><td>235</td></tr>
<tr><td>Citrobacter phage SH1</td><td>231</td></tr>
<tr><td>Citrobacter phage SH2</td><td>243</td></tr>
</table>

**Table S6: Prophage predictions using Phaster for the contigs containing the spanin gene**

| Organism | Contig accession | Predicted prophage position | Prophage score | Spanin position | Spanin accession | Spanin associated to prophage |
|---|---|---|---|---|---|---|
| Thalassobius gelatinovorus strain DSM 5887 | NZ_FOFW01000006 | 189621-213252 | intact | 208342..208716 | WP_058264408.1 | yes |
| Salinihabitans flavidus strain DSM 27842 | FODS01000069 | none, too short contig | | 1323..1676 | SEP27667 | unknown |
| Ruegeria mobilis strain DSM 23403 | NZ_FNNK01000005 | 157938-194421 | intact | 178515..178898 | WP_065332003 | yes |
| Ruegeria mobilis strain M41-2.2 | NZ_LNWW01000004 | 700765-722736 | incomplete | 721841..722224 | WP_065329472 | yes |
| Silicibacter sp. TM1040 | NC_008044.1 | 1376648-1397748 | intact | 1396955..1397338 | WP_011538643 | yes |
| Rhodovulum sp. MB263 | NZ_CP020384 | 862737-879872 | incomplete | 865553..865897 | WP_080615430 | yes |
| Rhodovulum sulfidophilum strain AB26 | MSYQ01000001 | many, not associated with the spanin | | 1904888..1905244 | OLS44559.1 | no |
| Phaeobacter sp. P97 | NZ_CP016364 | 1925407-1946347 | incomplete | 1945236..1945619 | WP_072504847 | yes |
| Phaeobacter inhibens strain S4Sm | NZ_LOHU01000025 | 4300-27092 | questionable | 5028..5411 | WP_061047696 | yes |
| Phaeobacter sp. S26 | NZ_JSWK01000008 | 125831-162508 | questionable | 140888..141271 | WP_040172280 | yes |
| Phaeobacter inhibens DSM 16374 | NZ_KI421498 | 1832819-1867875 | intact | 1854039..1854422 | WP_027247877 | yes |
| Phaeobacter inhibens DSM 17395 | NC_018290.1 | 1905561-1926725 | intact | 1925596..1925973 | WP_014880219 | yes |
| Leisingera sp. ANG-M7 | NZ_JWLI01000008.1 | 60402-97630 | intact | 77885..78262 | WP_052272404 | yes |

**Table S7: Environmental distribution of *Celeribacter marinus* IMCC 12053, based on the 16S rRNA blast hits in the NR Blast databases from NCBI, with minimum 99.0% identity.**

| Blast hit Accession | Isolation source | Country | coordinates | % nucleotide Identity | Alignment length | evalue |
|---|---|---|---|---|---|---|
| AJ391195.1 | Adriatic Sea | | | 100 | 1338 | 0 |
| AJ391196.1 | Adriatic Sea | | | 100 | 1360 | 0 |
| CP012023.1 | coastal surface seawater of the Yellow Sea | South Korea | | 100 | 1471 | 0 |
| AM990783.1 | sea water North Western Mediterranean Sea | France | 42.29 N 3.08 E | 99.858 | 1407 | 0 |
| AY697903.1 | seawater | Antarctica | | 100 | 1410 | 0 |
| NR_133717.1 | seawater, Yellow Sea | South Korea | | 99.853 | 1359 | 0 |
| HM140667.1 | toxigenic diatom Pseudo-nitzschia, Puget Sound | WA (Main Basin) | | 99.29 | 1409 | 0 |
| GU061042.1 | Yellow Sea intertidal beach | Korea | | 99.283 | 1394 | 0 |
| GU061048.1 | Yellow Sea intertidal beach | Korea | | 99.211 | 1394 | 0 |
| HM140672.1 | Puget Sound | WA (Main Basin) | | 99.219 | 1409 | 0 |
| HM140674.1 | Puget Sound | WA (Main Basin) | | 99.148 | 1409 | 0 |

**Table S8: Environmental distribution of *Lentibacter* sp. SH36, based on the 16S rRNA blast hits in the NR Blast databases from NCBI, with minimum 99.0% identity.**

| Blast hit Accession | Isolation source | Country | coordinates | % nucleotide Identity | Alignment length | evalue |
|---|---|---|---|---|---|---|
| AJ391182.1 | Adriatic Sea | | | 100 | 1429 | 0 |
| JQ269272.1 | estuary zone of Jiulong River | China | | 99.508 | 1422 | 0 |
| AF305498.1 | German bight | | | 99.502 | 1405 | 0 |
| AY701455.1 | Gymnodinium catenatum from Huon Estuary, | Tasmania (Australia) | | 99.71 | 1381 | 0 |
| DQ234098.2 | mangrove, Danshui river estuary | Northern Taiwan | | 99.862 | 1452 | 0 |
| DQ234152.2 | mangrove, Danshui river estuary | Northern Taiwan | | 99.931 | 1452 | 0 |

| | | | | | | |
|---|---|---|---|---|---|---|
| DQ234180.2 | mangrove, Danshui river estuary | Northern Taiwan | | 99.931 | 1452 | 0 |
| DQ234196.2 | mangrove, Danshui river estuary | Northern Taiwan | | 99.931 | 1452 | 0 |
| DQ234202.2 | mangrove, Danshui river estuary | Northern Taiwan | | 99.862 | 1452 | 0 |
| DQ234210.2 | mangrove, Danshui river estuary | Northern Taiwan | | 99.931 | 1452 | 0 |
| DQ234244.2 | mangrove, Danshui river estuary | Northern Taiwan | | 99.862 | 1452 | 0 |
| AY145564.1 | marine section of the Weser estuary | Germany | | 99.778 | 1350 | 0 |
| EU799040.1 | Newport Harbour, RI | USA | 41.486 N 71.351 W | 99.86 | 1428 | 0 |
| EU799044.1 | Newport Harbour, RI | USA | 41.486 N 71.351 W | 99.93 | 1428 | 0 |
| EU799171.1 | Newport Harbour, RI | USA | 41.486 N 71.351 W | 99.72 | 1428 | 0 |
| EU799468.1 | Newport Harbour, RI | USA | 41.486 N 71.351 W | 100 | 1403 | 0 |
| EU799546.1 | Newport Harbour, RI | USA | 41.486 N 71.351 W | 99.79 | 1428 | 0 |
| EU799658.1 | Newport Harbour, RI | USA | 41.486 N 71.351 W | 99.86 | 1428 | 0 |
| EU800046.1 | Newport Harbour, RI | USA | 41.486 N 71.351 W | 99.719 | 1426 | 0 |
| FJ882054.1 | North Sea, 2 m depth | | 54.420 N 6.480 E | 99.717 | 1415 | 0 |
| FJ154967.1 | ocean water from Bohai Bay | China | | 99.93 | 1422 | 0 |
| HM057661.1 | ocean water from the Yellow Sea | | | 99.93 | 1422 | 0 |
| JQ712107.1 | oil-contaminated seawater | | | 100 | 1422 | 0 |
| KJ094194.1 | polluted marine sediments | | | 99.848 | 1319 | 0 |
| EF659446.1 | Poole Harbour seawater | | | 99.854 | 1371 | 0 |
| HM591431.1 | pretreatment systems for seawater reverse osmosis process | South Korea | | 99.789 | 1423 | 0 |
| HM591461.1 | pretreatment systems for seawater reverse osmosis process | South Korea | | 99.93 | 1423 | 0 |
| JF514256.1 | Seawater from coast of Xiaomaidao Island, Qingdao | China | | 99.79 | 1426 | 0 |
| AM945553.1 | sea water, Adriatic sea | Italy | 44.69 N 12.52 E | 99.774 | 1329 | 0 |
| AM945571.1 | sea water, Adriatic sea | Italy | 44.69 N 12.52 E | 100 | 1269 | 0 |
| AM945577.1 | sea water, Adriatic sea | Italy | 44.69 N 12.52 E | 99.924 | 1315 | 0 |
| AM945578.1 | sea water, Adriatic sea | Italy | 44.69 N 12.52 E | 99.925 | 1325 | 0 |
| AM945580.1 | sea water, Adriatic sea | Italy | 44.69 N 12.52 E | 99.774 | 1330 | 0 |

| | | | | | | |
|---|---|---|---|---|---|---|
| AM945584.1 | sea water, Adriatic sea | Italy | 44.69 N 12.52 E | 99.696 | 1317 | 0 |
| AM945585.1 | sea water, Adriatic sea | Italy | 44.69 N 12.52 E | 99.699 | 1328 | 0 |
| EU930869.3 | sea water sample, Gwangyang Bay | South Korea | | 99.928 | 1385 | 0 |
| AB496659.1 | Seawater, Shizuoka, Shimoda, Oura Bay | Japan | | 100 | 1361 | 0 |
| FJ425223.1 | seawater | | | 99.783 | 1384 | 0 |
| FJ436731.1 | seawater, coast of Qingdao | China | 36.0276 N 120.1846 E | 99.925 | 1328 | 0 |
| JQ195110.1 | seawater; next to dolphin A, San Diego Bay | USA: San Diego, CA | | 99.844 | 1281 | 0 |
| JQ195194.1 | seawater; next to dolphin A, San Diego Bay | USA: San Diego, CA | | 99.844 | 1283 | 0 |
| JQ195765.1 | seawater; next to dolphin C, San Diego Bay | USA: San Diego, CA | | 99.922 | 1281 | 0 |
| JQ195767.1 | seawater; next to dolphin C, San Diego Bay | USA: San Diego, CA | | 100 | 1281 | 0 |
| JQ196782.1 | seawater; next to dolphin E, San Diego Bay | USA: San Diego, CA | | 100 | 1281 | 0 |
| JQ712031.1 | surface seawater offshore Qingdao | China | | 99.367 | 1422 | 0 |
| JQ712040.1 | surface seawater offshore Qingdao | | | 99.93 | 1422 | 0 |
| JQ712053.1 | surface seawater offshore Qingdao | | | 99.93 | 1422 | 0 |
| JQ712057.1 | surface seawater offshore Qingdao | | | 100 | 1422 | 0 |
| NR_108333.1 | coastal region of Qingdao | China | 36.027N 120.184E | 100 | 1370 | 0 |

**Table S9: Environmental distribution of *Roseobacter* sp. GAI101, based on the 16S rRNA blast hits in the NR Blast databases from NCBI, with minimum 99.0% identity.**

| Blast hit Accession | Isolation source | Country | coordinates | % nucleotide Identity | Alignment length | evalue |
|---|---|---|---|---|---|---|
| JX529099.1 | 500m depth water samples filtered on 0.22 micron filter paper; derived from the Southern ocean iron fertilization experiment (LOHAFEX) | | 47.9533 S 15.1437 W | 99.561 | 1367 | 0 |

| | | | | | | |
|---|---|---|---|---|---|---|
| JX529112.1 | 500m depth water samples filtered on 0.22 micron filter paper; derived from the Southern ocean iron fertilization experiment (LOHAFEX) | | 47.9533 S 15.1437 W | 99.634 | 1367 | 0 |
| JX529220.1 | 500m depth water samples filtered on 0.22 micron filter paper; derived from the Southern ocean iron fertilization experiment (LOHAFEX) | | 47.9533 S 15.1437 W | 99.561 | 1367 | 0 |
| JX531118.1 | 500m depth water samples filtered on 0.22 micron filter paper; derived from the Southern ocean iron fertilization experiment (LOHAFEX) | | 47.5015 S 15.4450 W | 99.561 | 1367 | 0 |
| JX531139.1 | 500m depth water samples filtered on 0.22 micron filter paper; derived from the Southern ocean iron fertilization experiment (LOHAFEX) | | 47.5015 S 15.4450 W | 99.635 | 1368 | 0 |
| JX531386.1 | 500m depth water samples filtered on 0.22 micron filter paper; derived from the Southern ocean iron fertilization experiment (LOHAFEX) | | 47.5015 S 15.4450 W | 99.415 | 1368 | 0 |
| AJ278782.1 | Antarctic seawater isolate | | | 99.571 | 1399 | 0 |
| EU016167.1 | Arctic deep-sea sediment | | | 99.719 | 1424 | 0 |
| FJ889522.1 | Arctic Ocean | | | 99.58 | 1430 | 0 |
| FJ889535.1 | Arctic Ocean | | | 99.579 | 1424 | 0 |
| FJ889536.1 | Arctic Ocean | | | 99.579 | 1424 | 0 |
| FJ889527.1 | Arctic Ocean | | | 99.51 | 1430 | 0 |
| FJ889528.1 | Arctic Ocean | | | 99.511 | 1431 | 0 |
| FJ889530.1 | Arctic Ocean | | | 99.368 | 1424 | 0 |
| FJ889531.1 | Arctic Ocean | | | 99.28 | 1388 | 0 |
| FJ889542.1 | Arctic Ocean | | | 99.298 | 1424 | 0 |
| EU365544.1 | Arctic seawater | | | 99.58 | 1430 | 0 |
| EU365589.1 | Arctic seawater | | | 99.65 | 1430 | 0 |
| JX525417.1 | chlrophyll maxima (~30m) water samples filtered on 0.22 micron filter paper; derived from the Southern ocean iron fertilization experiment (LOHAFEX) | | 48.0329 S 15.7856 W | 99.561 | 1367 | 0 |

| | | | | | | |
|---|---|---|---|---|---|---|
| JX525429.1 | chlrophyll maxima (~30m) water samples filtered on 0.22 micron filter paper; derived from the Southern ocean iron fertilization experiment (LOHAFEX) | | 48.0329 S 15.7856 W | 99.707 | 1367 | 0 |
| JX525500.1 | chlrophyll maxima (~30m) water samples filtered on 0.22 micron filter paper; derived from the Southern ocean iron fertilization experiment (LOHAFEX) | | 48.0329 S 15.7856 W | 99.489 | 1370 | 0 |
| JX525579.1 | chlrophyll maxima (~30m) water samples filtered on 0.22 micron filter paper; derived from the Southern ocean iron fertilization experiment (LOHAFEX) | | 48.0329 S 15.7856 W | 99.634 | 1367 | 0 |
| JX527826.1 | chlrophyll maxima (~30m) water samples filtered on 0.22 micron filter paper; derived from the Southern ocean iron fertilization experiment (LOHAFEX) | | 47.9533 S 15.1437 W | 99.488 | 1368 | 0 |
| FN377731.1 | clay-like sticky sediment with cylindrical plants/weeds on surface less than KB3 | Svalbard, Kongsfjord region | 78.59 N 11.56 E | 99.52 | 1459 | 0 |
| KJ939484.1 | continental shelf sediments of Bay of Bengal | | | 99.56 | 1364 | 0 |
| NR_043547.1 | East Sea | Korea | | 99.217 | 1404 | 0 |
| FR684971.1 | marine biome, fjord, coastal water | Norway, Raunefjord | 60.27 N 5.22 E | 99.508 | 1423 | 0 |
| KJ475186.1 | marine sediment, Ross Sea, Antarctica | | 76°06'17.10''S/ 169°12'45.36''E - 76°41'03.60"S/ 169°11'30.66"E | 99.634 | 1365 | 0 |
| AB733557.1 | Nansei-Shotō Trench off Miyako Island, Okinawa Prefecture, southern Japan | | | 99.719 | 1421 | 0 |
| AY573043.1 | Ny-Alesund, Svalbard, Norway | arctic | 79° N, 12° E | 99.579 | 1426 | 0 |
| AY697915.1 | seawater | Antarctica | | 99.37 | 1428 | 0 |
| GQ358930.1 | seawater from Kongsfjorden, Spitsbergen | Norway | | 99.579 | 1424 | 0 |
| AF007257.2 | seawater, Skidaway River | | | 99.929 | 1414 | 0 |

| | | | | | | |
|---|---|---|---|---|---|---|
| AY794211.1 | Soil from Ushuaia | Argentina | 54°80' S and 68°13' W | 99.37 | 1429 | 0 |
| JX310158.1 | Southern Ocean | | 47–48◦ South and 15–17◦ West | 99.04 | 1458 | 0 |
| GU584137.1 | water sample | Antarctica | 47–48°S and 15–17°W | 99.588 | 1458 | 0 |
| EF471650.1 | whole surface water from Chesapeake Bay | | 39.13 N 76.33 W | 99.719 | 1423 | 0 |
| AY902203.1 | | | | 99.489 | 1369 | 0 |
| JX529304.1 | 500m depth water samples filtered on 0.22 micron filter paper; derived from the Southern ocean iron fertilization experiment (LOHAFEX) | | 47.9533 S 15.1437 W | 99.122 | 1367 | 0 |

**Table S10: Environmental distribution of *Roseobacter* sp. SIO, based on the 16S rRNA blast hits in the NR Blast databases from NCBI, with minimum 99.6% identity.**

| Blast hit Accession | Isolation source | Country | coordinates | % nucleotide Identity | Alignment length | evalue |
|---|---|---|---|---|---|---|
| JX864948.1 | 5m depth, the Gulf of Trieste, northernAdriatic Sea, NE Mediterranean | | | 99.692 | 325 | 1.92E-166 |
| JX865037.1 | 5m depth, the Gulf of Trieste, northernAdriatic Sea, NE Mediterranean | | | 99.692 | 325 | 1.92E-166 |
| JX865038.1 | 5m depth, the Gulf of Trieste, northernAdriatic Sea, NE Mediterranean | | | 99.692 | 325 | 1.92E-166 |
| JX865039.1 | 5m depth, the Gulf of Trieste, northernAdriatic Sea, NE Mediterranean | | | 99.692 | 325 | 1.92E-166 |
| JX865040.1 | 5m depth, the Gulf of Trieste, northernAdriatic Sea, NE Mediterranean | | | 99.692 | 325 | 1.92E-166 |
| JX865041.1 | 5m depth, the Gulf of Trieste, northernAdriatic Sea, NE Mediterranean | | | 99.692 | 325 | 1.92E-166 |
| JX865044.1 | 5m depth, the Gulf of Trieste, northernAdriatic Sea, NE Mediterranean | | | 99.688 | 320 | 1.16E-163 |
| JX865047.1 | 5m depth, the Gulf of Trieste, northernAdriatic Sea, NE Mediterranean | | | 99.692 | 325 | 1.92E-166 |
| JX865055.1 | 5m depth, the Gulf of Trieste, northernAdriatic Sea, NE Mediterranean | | | 99.692 | 325 | 1.92E-166 |
| JX865056.1 | 5m depth, the Gulf of Trieste, northernAdriatic Sea, NE Mediterranean | | | 99.692 | 325 | 1.92E-166 |
| JX865060.1 | 5m depth, the Gulf of Trieste, northernAdriatic Sea, NE Mediterranean | | | 99.692 | 325 | 1.92E-166 |
| JX865061.1 | 5m depth, the Gulf of Trieste, northernAdriatic Sea, NE Mediterranean | | | 99.692 | 325 | 1.92E-166 |
| AB703474.1 | coastal hot spring, Kagoshima, Ibusuki hot spring | Japan: | | 99.692 | 325 | 1.92E-166 |
| AB703500.1 | coastal hot spring, Kagoshima, Ibusuki hot spring | Japan: | | 99.692 | 325 | 1.92E-166 |

| | | | | | | |
|---|---|---|---|---|---|---|
| AF007250.1 | coastal seawater, Sapelo Island | | | 99.686 | 318 | 1.50E-162 |
| LT549272.1 | corrosion biofilm Boothbay Harbor | USA:Maine | 43.8443 N 69.6409 W | 99.692 | 325 | 1.92E-166 |
| LT549313.1 | corrosion biofilm Boothbay Harbor | USA:Maine | 43.8443 N 69.6409 W | 99.692 | 325 | 1.92E-166 |
| LT549329.1 | corrosion biofilm Boothbay Harbor | USA:Maine | 43.8443 N 69.6409 W | 99.692 | 325 | 1.92E-166 |
| LT549343.1 | corrosion biofilm Boothbay Harbor | USA:Maine | 43.8443 N 69.6409 W | 99.692 | 325 | 1.92E-166 |
| DQ234090.2 | Danshui river estuary, mangrove | Northern Taiwan | | 99.692 | 325 | 1.92E-166 |
| DQ234137.2 | Danshui river estuary, mangrove | Northern Taiwan | | 99.692 | 325 | 1.92E-166 |
| DQ234157.2 | Danshui river estuary, mangrove | Northern Taiwan | | 99.692 | 325 | 1.92E-166 |
| DQ234162.2 | Danshui river estuary, mangrove | Northern Taiwan | | 99.692 | 325 | 1.92E-166 |
| DQ234224.2 | Danshui river estuary, mangrove | Northern Taiwan | | 99.692 | 325 | 1.92E-166 |
| KC006265.1 | estuary in middle of Jiulong River | China | 24.26 N 117.127 E | 99.69 | 323 | 2.48E-165 |
| KC006295.1 | estuary in middle of Jiulong River | China | 24.26 N 117.129 E | 99.692 | 325 | 1.92E-166 |
| KT275136.1 | Gut of Olive Flounder Paralichthys olivaceus | | | 99.692 | 325 | 1.92E-166 |
| EF215736.1 | inert artificial surfaces submerged in marine water on Qingdao coast | China | | 99.692 | 325 | 1.92E-166 |
| AY712068.1 | lon=81.2699W, lat=31.3929N; surface water collected on Jul 18, 2001, Sapelo Island Microbial Observatory Dean Creek Marsh sampling site | USA: Georgia | | 99.692 | 325 | 1.92E-166 |
| AY937021.1 | marine sediment Nagasaki | Japan: | | 99.692 | 325 | 1.92E-166 |
| AY941090.1 | marine sediment Nagasaki | Japan: | | 99.692 | 325 | 1.92E-166 |
| KC462965.1 | nature reserve Kullaberg | Sweden | | 99.692 | 325 | 1.92E-166 |
| KC462968.1 | nature reserve Kullaberg | Sweden | | 99.692 | 325 | 1.92E-166 |
| EU799217.1 | Newport Harbour, RI | USA | 41.486 N 71.351 W | 99.692 | 325 | 1.92E-166 |
| EU799316.1 | Newport Harbour, RI | USA | 41.486 N 71.351 W | 99.692 | 325 | 1.92E-166 |
| EU799841.1 | Newport Harbour, RI | USA | 41.486 N 71.351 W | 99.692 | 325 | 1.92E-166 |
| JQ432672.1 | Northern Adriatic Sea, NE Mediterranean | | | 99.692 | 325 | 1.92E-166 |

| | | | | | | |
|---|---|---|---|---|---|---|
| KF185598.1 | Northern Adriatic Sea: Gulf of Trieste,coastal seawater sample 10m depth | | | 99.692 | 325 | 1.92E-166 |
| AY576710.1 | northwestern Mediterranean Sea | | | 99.692 | 325 | 1.92E-166 |
| GQ347802.1 | Saanich Inlet, 10 m depth | | 48.5883 N 123.5037 W | 99.692 | 325 | 1.92E-166 |
| AF190747.1 | Scripps Institution of Oceanography Pier | | | 100 | 325 | 4.13E-168 |
| U64005.1 | Scripps Pier | | | 99.692 | 325 | 1.92E-166 |
| AM945581.1 | sea water | Italy:Adriatic sea | 44.69 N 12.52 E | 99.692 | 325 | 1.92E-166 |
| HQ203887.1 | seawater | | | 99.692 | 325 | 1.92E-166 |
| HM921237.1 | seawater, Kiel Fjord | | 54°27’4N, 10°12’E | 99.692 | 325 | 1.92E-166 |
| FJ161222.1 | Shandong coast | China | | 99.69 | 323 | 2.48E-165 |
| FJ161239.1 | Shandong coast, China | | | 99.692 | 325 | 1.92E-166 |
| EF491274.1 | steel surfaces immerged in marine waterof Qingdao Coast | | | 99.692 | 325 | 1.92E-166 |
| AB362414.1 | surface seawater | Japan:Niigata, Urahama coast | | 99.692 | 325 | 1.92E-166 |
| HE981601.1 | surface seawater | Spain:Mallorca Island, Palma Harbour | | 99.688 | 321 | 3.21E-164 |
| KP198349.1 | surface seawater | USA: Cape Cod Bay | 41.744 N 70.219 W | 99.692 | 325 | 1.92E-166 |
| GU061006.1 | Yellow Sea intertidal beach | Korea | | 99.692 | 325 | 1.92E-166 |
| GU061090.1 | Yellow Sea intertidal beach | Korea | | 99.692 | 325 | 1.92E-166 |
| GU061106.1 | Yellow Sea intertidal beach | Korea | | 99.692 | 325 | 1.92E-166 |
| AF365990.1 | | USA: off La Jolla, CA | | 99.692 | 325 | 1.92E-166 |
| AY536559.1 | | | | 99.692 | 325 | 1.92E-166 |
| JN653206.1 | | USA: Bayboro Harbor, Florida | 27.76 N 82.63 W | 99.692 | 325 | 1.92E-166 |

**Table S11: Prophage prediction using Phaster for the contigs containing the glutaredoxin gene**

| Organism | Contig accession | Predicted prophage position | Prophage score | Glutaredoxin position | Glutaredoxin accession | Glutaredoxin associated to prophage |
|---|---|---|---|---|---|---|
| Rhizobium sp. Leaf383 | NZ_LMQD01000008 | 1407-72420 | intact | 39353..39715 | WP_082506598 | yes |
| Sinorhizobium sp. LM21 (plasmid) | KM659098 | 11856-47482 | questionable | 26157..26360 | AJW30150 | yes |
| Hepatospora eriocheir strain GB1 | LVKB01000119 | none | | 3861..4241 | ORD96177 | |
| Hepatospora eriocheir strain GB2 | LVKB01000120 | none | | 3003..3317 | ORD96175 | |

**Table S12: Prophage prediction using Phaster for the contigs containing the RNR genes**

| Organism | Contig accession | Predicted prophage position | Prophage score | RNR position | RNR accession | RNR associated to prophage |
|---|---|---|---|---|---|---|
| Aminobacter sp. J41 | NZ_JAGL01000009.1 | 44041-86811 | intact | 72083-74071 | WP_024847845 | yes |
| Inquilinus limosus MP06 | NZ_JANX01000001.1 | 32458-86185 | questionable | 47409..49382 | WP_034830439 | yes |
| Azorhizobium caulinodans ORS 571 | NC_009937.1 | 4097072-4138015 | questionable | 4122652..4124646 | WP_012172112 | yes |
| Microvirga flocculans ATCC BAA-817 | NZ_JAEA01000002.1 | 156454-179652 | incomplete | 163007..164998 | WP_027314944 | yes |
| Streptomyces sp. RTd22 | NZ_CP015726.1 | 8923271-8946147 | incomplete | 8923015..8924970 | WP_063728949.1 | yes |
| Streptomyces sp. CB03238 | NZ_NBCN01000010 | 231547-250436 | incomplete | 227661..229613 | WP_084899922 | in vicinity |
| Streptomyces sp. NRRL F-4489 | NZ_LLZI01000124 | 13535-24084 | incomplete | 19443..21374 | WP_066977110 | yes |
| Streptomyces rimosus subsp. rimosus strain NRRL WC-3904 | NZ_JOCQ01000004 | 101674-119267 | incomplete | 111002..112933 | WP_050514495 | yes |

| | | | | | | |
|---|---|---|---|---|---|---|
| Solirubrobacter sp. URHD0082 | NZ_AUEK01000007 | 344542-357687 | incomplete | 307203..309158 | WP_051323998 | in vicinity |
| Labrenzia alexandrii DFL-11 | EQ973124 | 3254-38475 | questionable | 15955..18009 | EEE42840 | yes |
| Methylobacterium sp. Leaf361 | NZ_LMPY01000023.1 | 581118-616465 | questionable | 594636..596663 | WP_082557943 | yes |
| Methylobacterium aquaticum plasmid pMaq22A_3p | NZ_AP014707 | 65911-85601 | incomplete | 83571..85601 | WP_060851403 | yes |
| Marinobacter similis strain A3d10 | CP007151.1 | 3645543-3690844 | intact | 3653541..3655502 | AHI29708.1 | yes |
| Protochlamydia amoebophila strain EI2 DB44_AM | NZ_JSAN01000012.1 | none | | 231..2243 | WP_039355956 | no |
| Gonium pectorale isolate NIES-2863 | LSYV01000168 | none | | 31484..37421 | KXZ42284.1 | no |
| Volvox carteri f. nagariensis | NZ_JOCQ01000004.1 | none | | <195692..>202991 | XP_002955713 | no |
| Criblamydia sequanensis | NZ_CCEJ010000007 | none | | 134416..136428 | WP_041017866 | no |
| Parachlamydia acanthamoebae strain Bn9 | NZ_BAWW01000003 | none | | 161269..163278 | WP_006342605 | no |
| Parachlamydia acanthamoebae strain OEW1 DB43_DW | NZ_JSAM01000017 | none | | 15051..17060 | WP_006342605 | no |
| Parachlamydia acanthamoebae UV7 | NC_015702 | none | | 895276..897285 | WP_006342605 | no |
| Parachlamydia acanthamoebae str. Hall's coccus | NZ_ACZE01000088 | none | | 17369..19378 | WP_006342605 | no |
| Estrella lausannensis | CWGJ01000019 | none | | 124560..126572 | CRX38802 | no |
| Candidatus Rubidus massiliensis | CCSC01000001 | 1811588-1828589 | incomplete | 1412888..1414906 | CDZ80757 | no |
| Waddlia chondrophila | NC_014225 | none | | 368424..370439 | WP_013181434 | no |
| Waddlia chondrophila 2032/99 | FR872643 | none | | 26560..28614 | CCB90950 | no |

| | | | | | | |
|---|---|---|---|---|---|---|
| Neochlamydia sp. TUME1 | NZ_JRXI01000032 | none | | 32033..34045 | WP_039383590 | no |
| Neochlamydia sp. S13 | NZ_BASK01001259 | none | | 36271..38283 | WP_042242257 | no |
| Neochlamydia sp. EPS4 | NZ_JSDQ01000098 | none | | 4026..6038 | WP_044882767 | no |
| Parachlamydia sp. C2 | NZ_FCNU01000032.1 | none | | 238258..240270 | WP_068471381 | no |
| Candidatus Protochlamydia amoebophila UWE25 | NC_005861 | none | | 178729..180741 | WP_011174661 | no |
| Candidatus Protochlamydia sp. W-9 | NZ_BCPZ01000156.1 | none | | 50658..52670 | WP_075883166 | no |
| Candidatus Protochlamydia sp. R18 | NZ_BASL01000738 | none | | 1527..3539 | WP_042281843 | no |

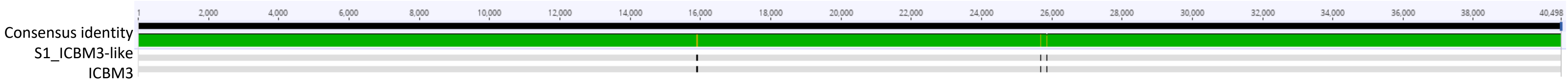


**Figure S1: Pairwise genome comparison between ICBM3 and the ICBM3-like genome assembled from the S1 enrichment. In the alignment field, the differences are marked in black. In the consensus identity field, the presence of mismatches is signaled in orange.**

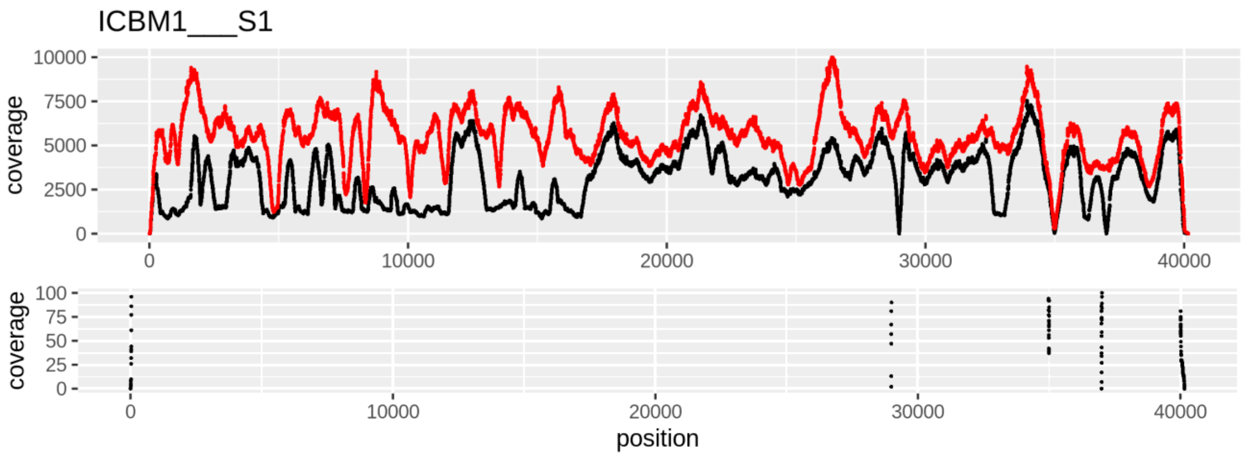
ICBM1___S1
coverage
position

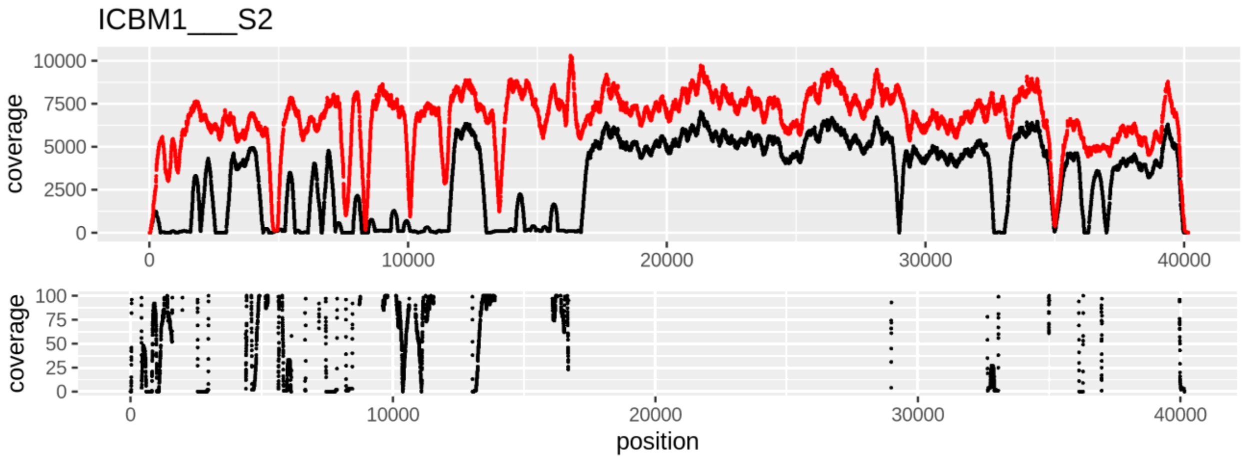
ICBM1___S2
coverage
position

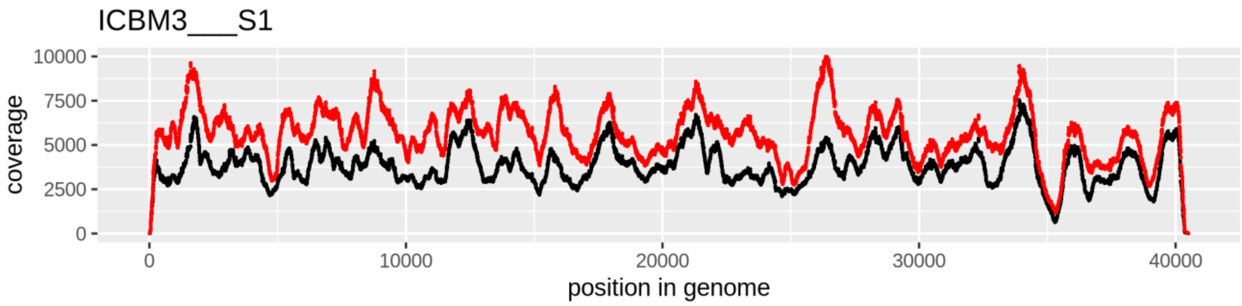
ICBM3___S1
coverage
position in genome

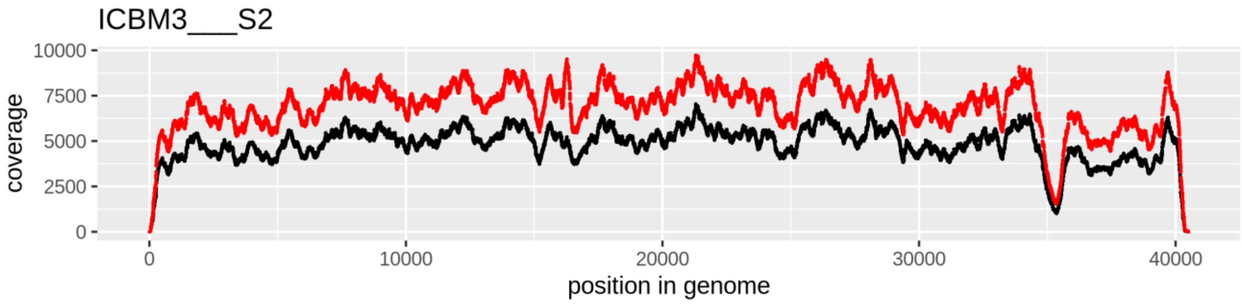
ICBM3___S2
coverage
position in genome

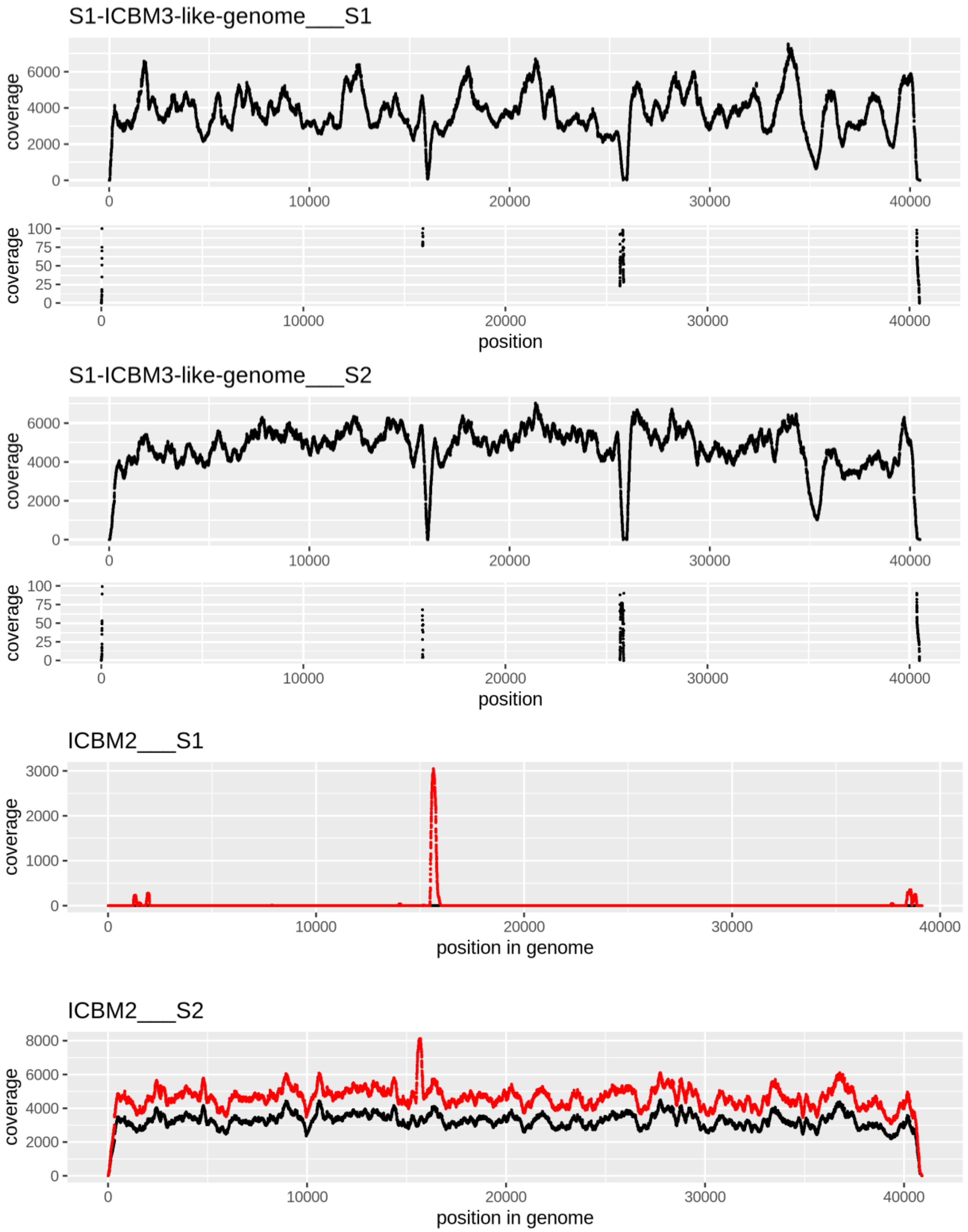


**Figure S2: Plots showing the read coverage along the phage genomes for the S1 and S2 phage enrichments. In black – coverage of reads with 100% identity. In red – coverage of reads with >95% identity. The small differences between ICBM3 and the ICBM3-like genome from S1 enrichment could be due to sequencing errors, as the coverage in the variable regions dropped sharply for the genome retrieved from S1, but not for ICBM3 (see Figure S1).**

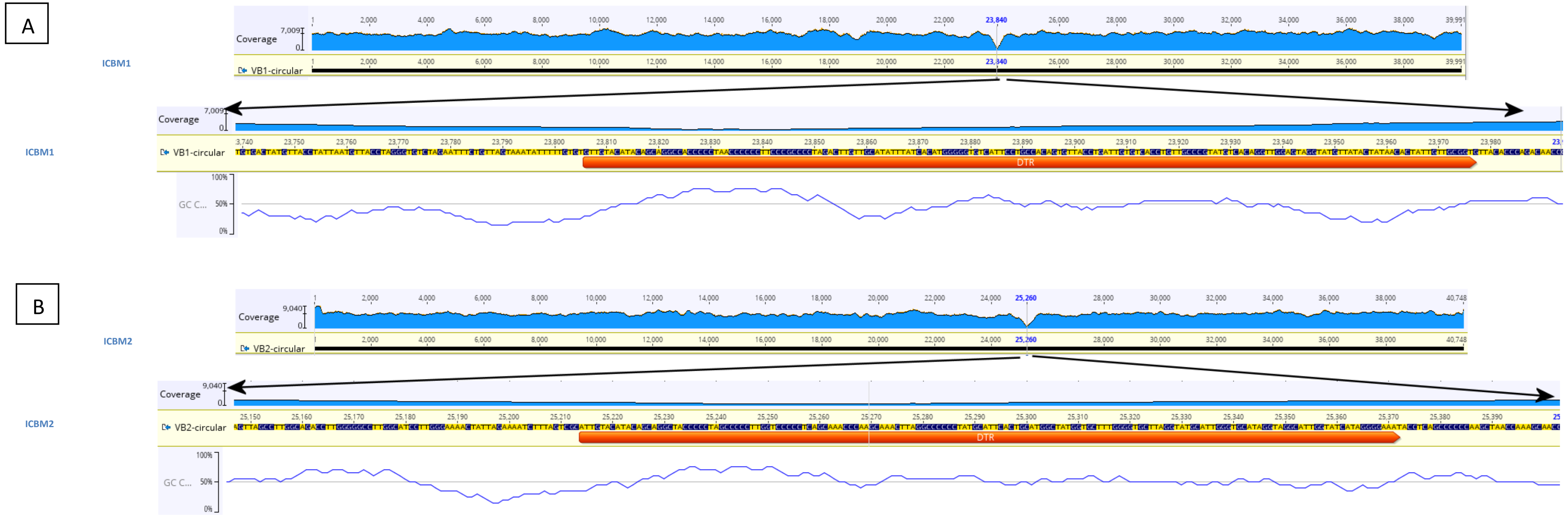


**Figure S3: Illumina short read sequencing, distribution of reads along the assembled contigs for ICBM1 (A) and ICBM2 (B) phages. The position of the drop in coverage is indicated in blue numbers. The magnifications show the region corresponding to the drop in coverage, with the corresponding %GC graph (blue line) and the DTR positions (as determined from PacBio read coverage) marked in red.**

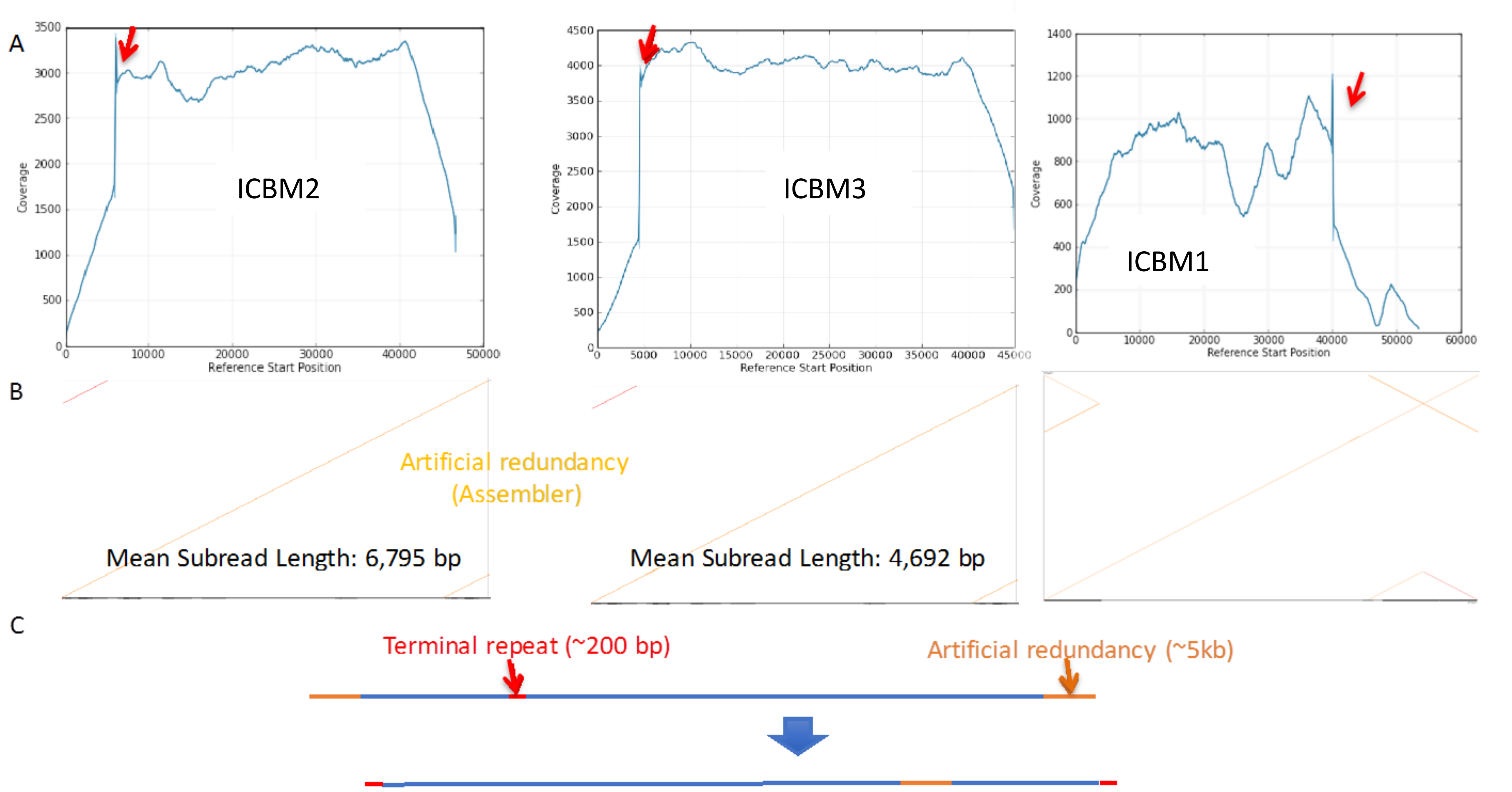


**Figure S4. A. Coverage plots (window = 100 bp) of long read genome assemblies of both phages ICBM1 and ICBM2 contained within enrichment. Distinct coverage spikes of about 100 bp can be observed at position 5kb (terminal redundancies, red arrows).**
**B. Artificial redundancies at the end of the contigs (shown here as dotplots) are produced by the genome assembler usually at the size of the mean subread length suggesting a circular genome structure.**

**C. Delineation of the final phage genome structure applying the "linearization" process. The artificial redundancy (yellow) is removed and the terminal repeat (red) placed twice at the end of the phage genomes**

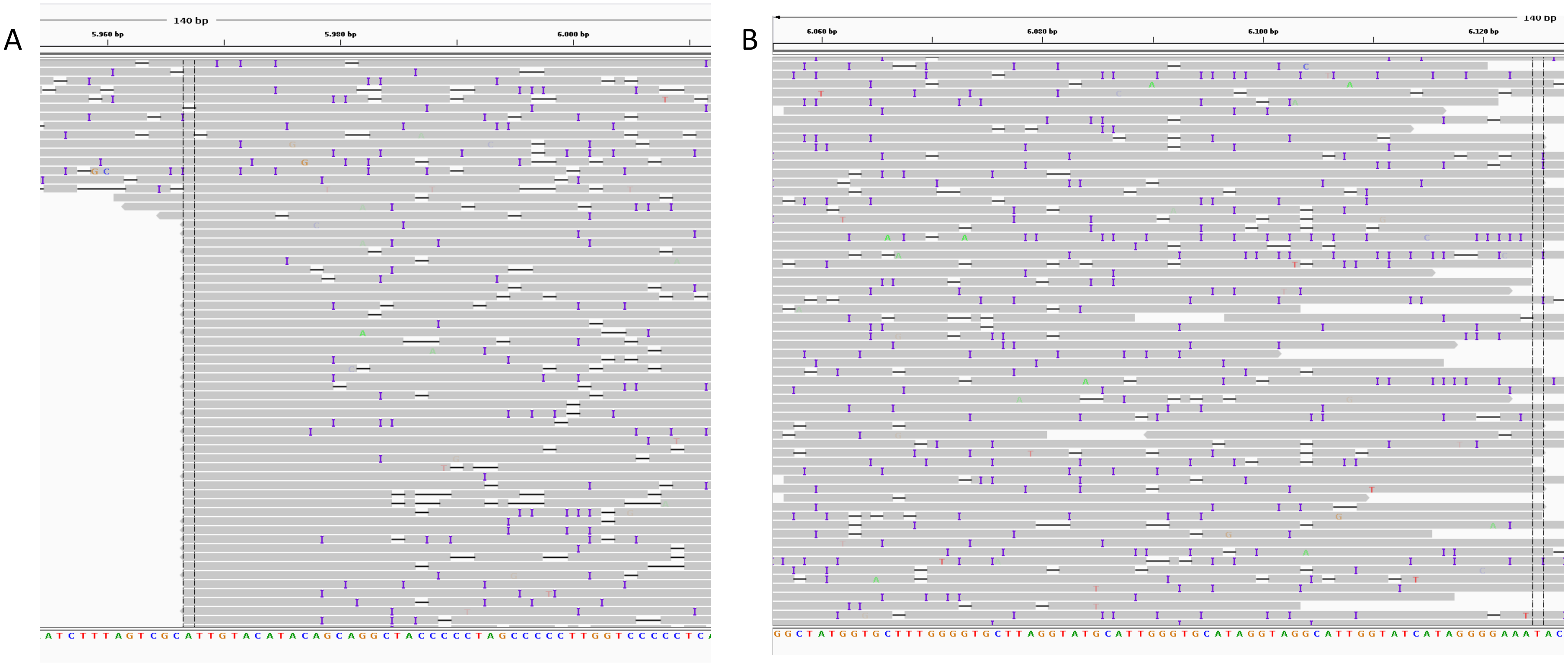


**Figure S5. Distinct start (A) and end positions (B) of a linear phage genome as retrieved from a visualization of a long read genome mapping by IGV.**

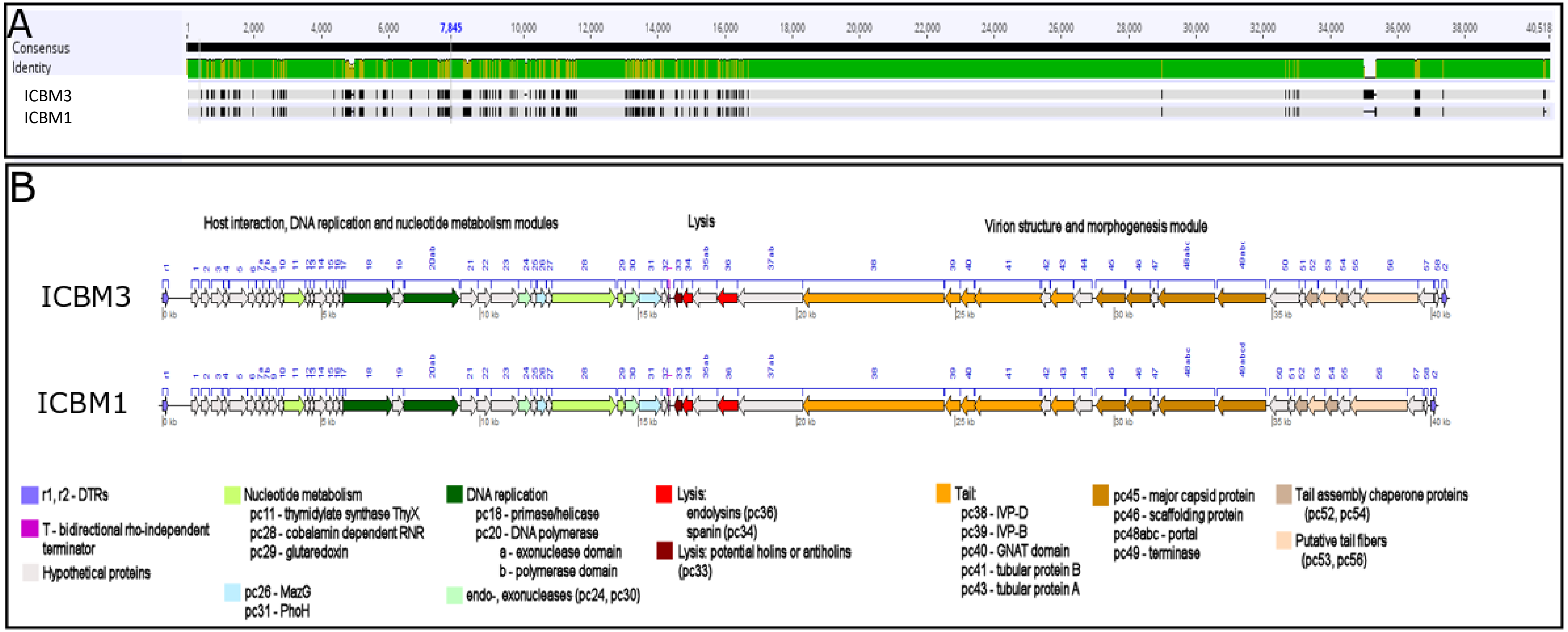


**Figure S6: Genome comparison of ICBM1 and ICBM3 phages: A) showing the location of mismatches and deletions and B) showing the predicted genes.**

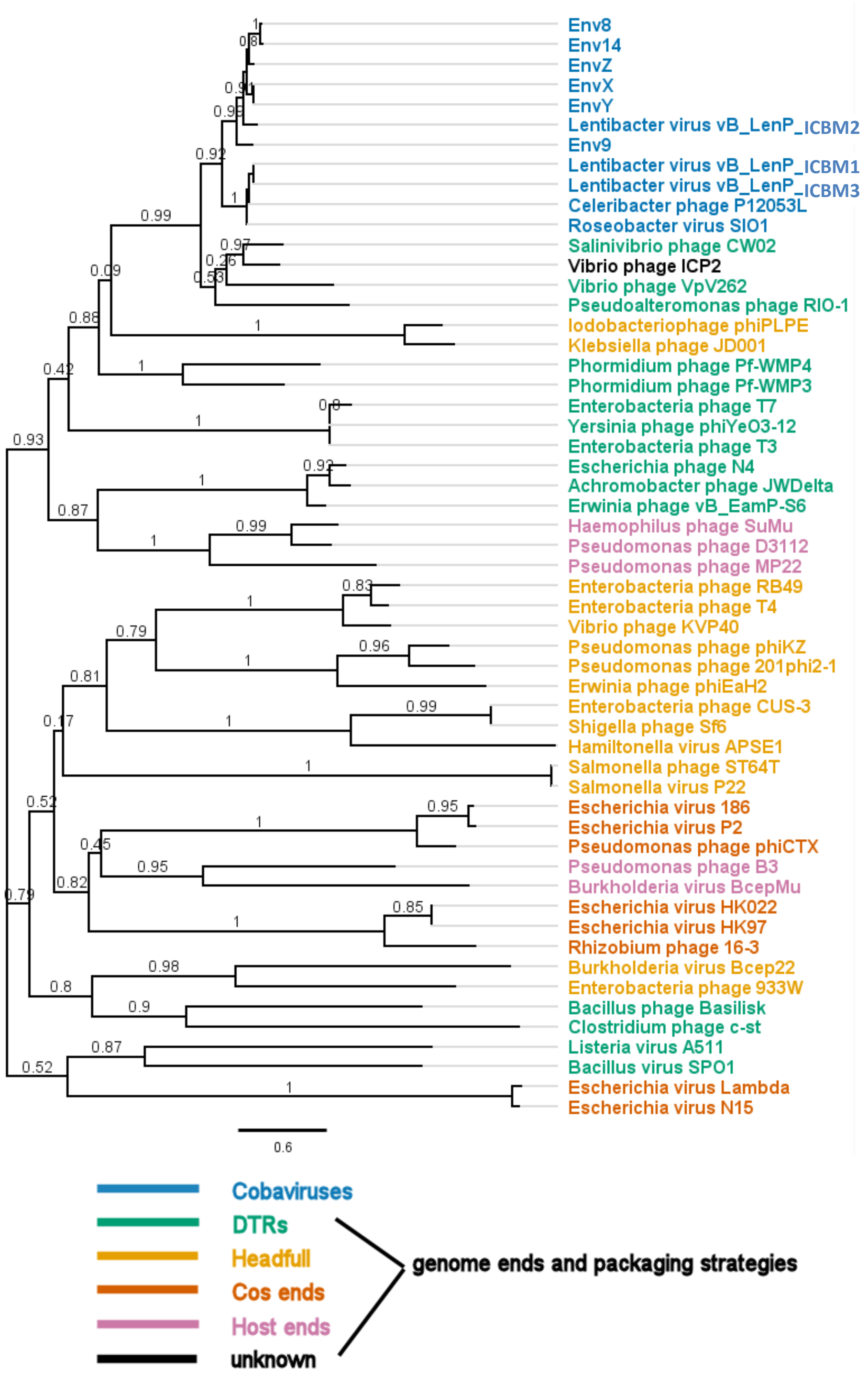


**Figure S7: Phylogenetic analysis of the Terminase large subunit from cobaviruses and other phages with known genome ends and packaging strategies. The evolutionary history was inferred using the approximately-maximum-likelihood method implemented in FastTree 2.1.5. The node labels represent Fast Tree support values. The tree is drawn to scale, with branch lengths measured in number of substitutions per site. The tree is unrooted.**

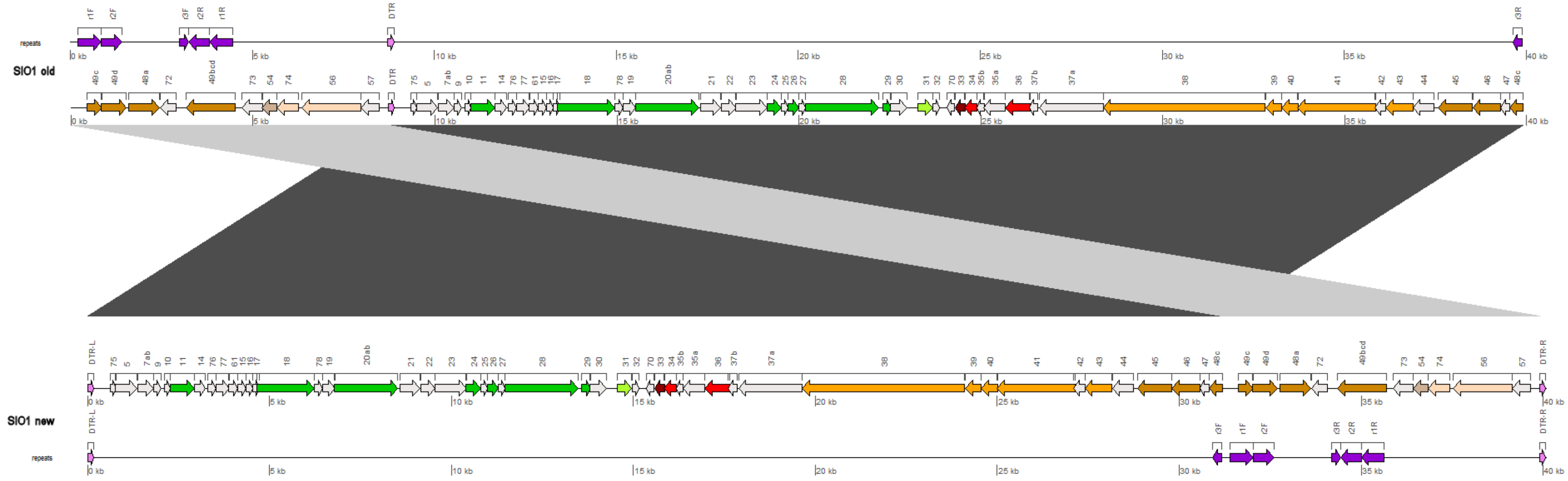


**Figure S8: Genomic maps of the SIO1 phage, before and after genome end correction. Purple arrows: position of the inverted repeats. Pink arrows: position of DTRs.**

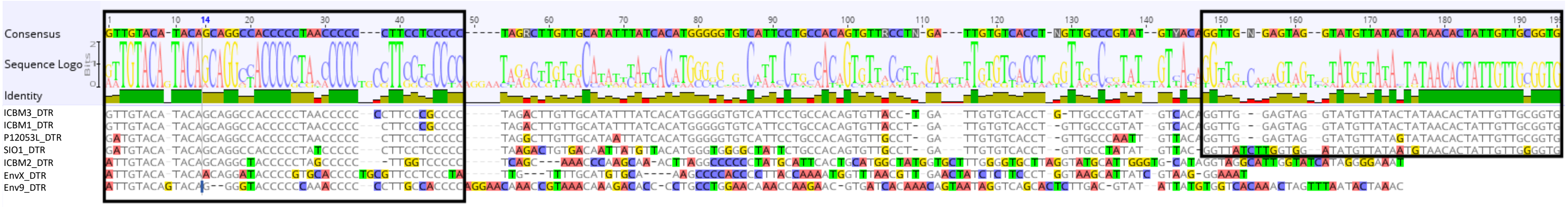


**Figure S9: Multiple alignments of genomic ends from all cobaviruses with complete genome.**

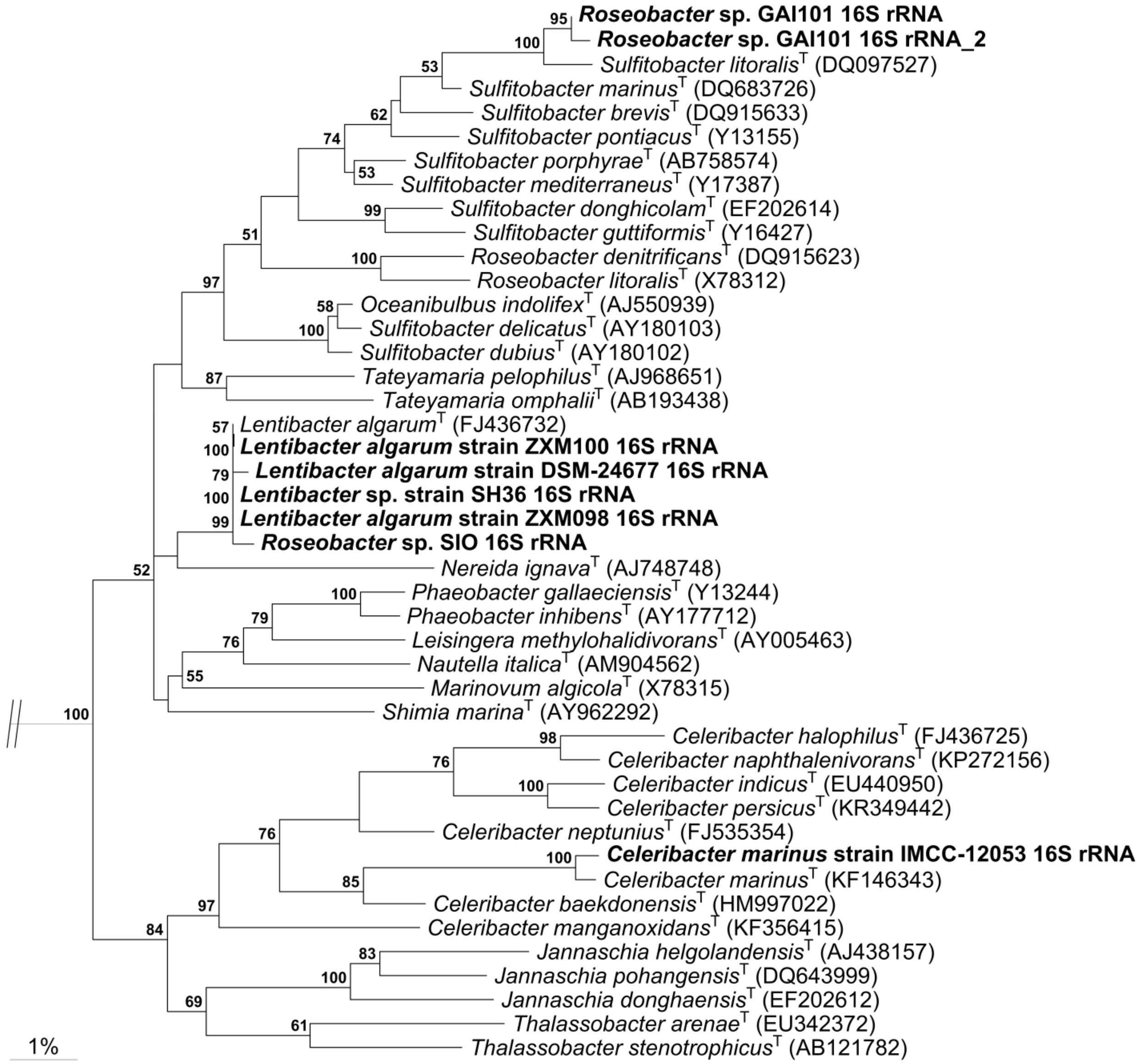


**Figure S10: Neighbour joining tree based on 16S rRNA gene similarity showing the phylogenetic affiliation of bacterial hosts analysed in this study (bold) within the *Rhodobacteraceae*. Sequences of type material (>1300bp) were used to construct the backbone tree. Only bootstrap values ≥50% (derived from 1500 replicates) are shown. Selected sequences related to *Gammaproteobacteria* were used as outgroup to define the root of the tree (not shown). GenBank accession numbers are given in parentheses. Scale bar indicates percentage of sequence divergence.**

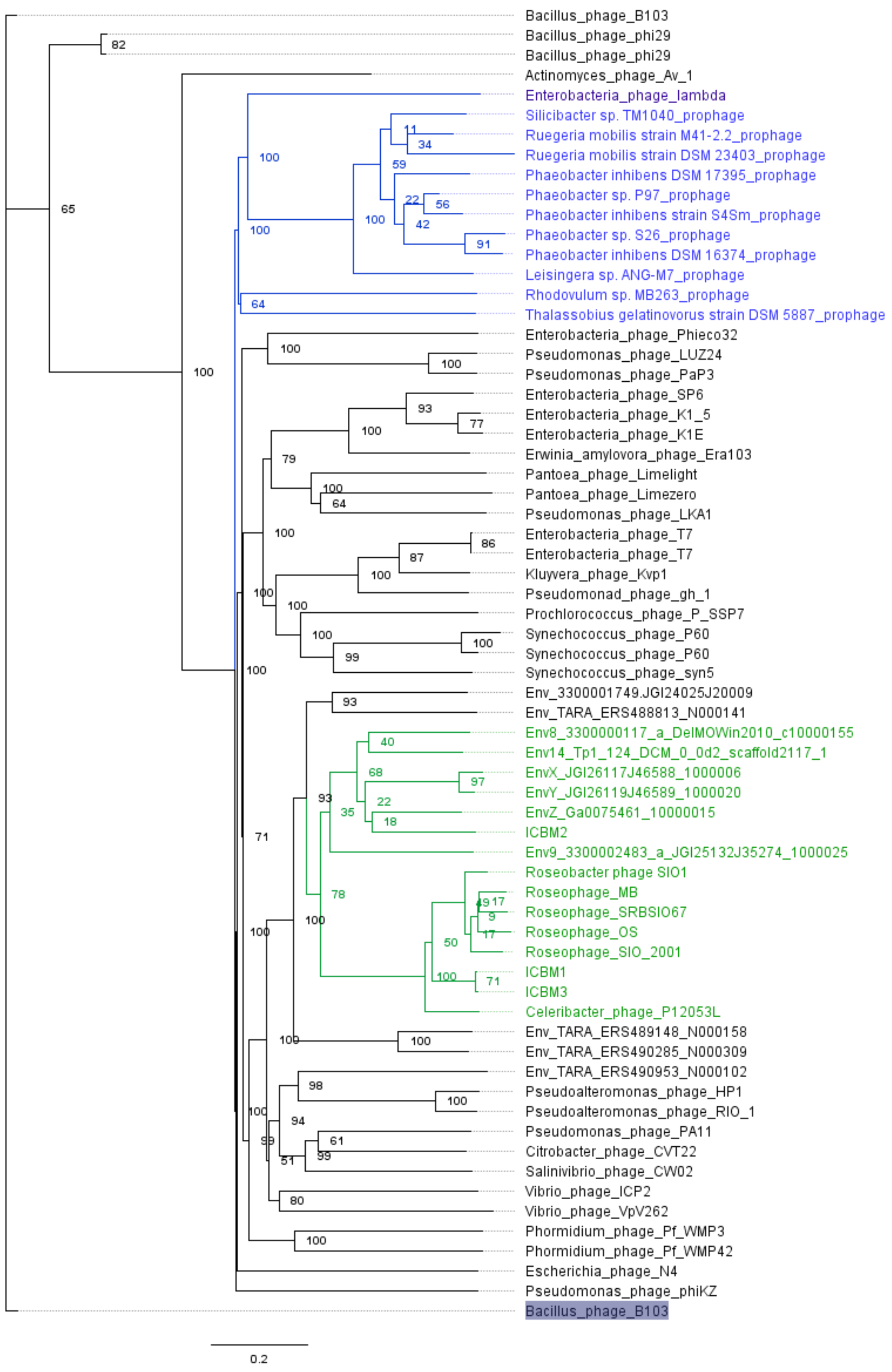


**Figure S11: Phylogenetic positioning of the Cobaviruses (green font) and spanin containing prophages from *Rhodobacteraceae* (blue font). The whole-genome-based phylogeny was inferred using the Genome-BLAST Distance Phylogeny method implemented in the VICTOR web service, using the amino acid data.**

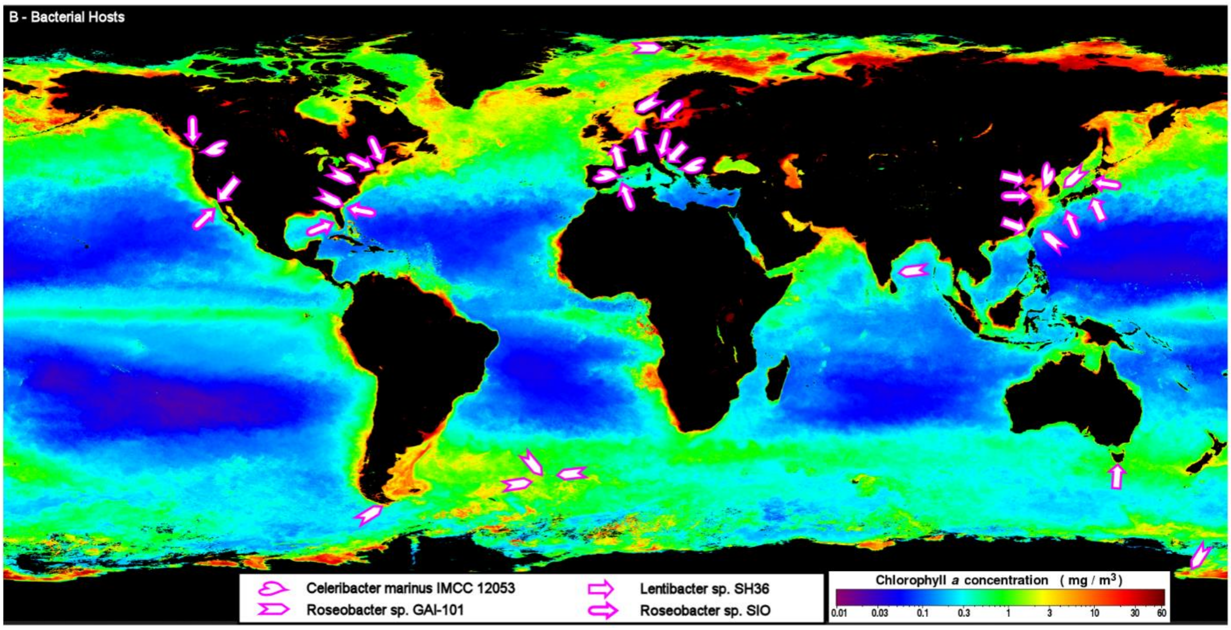


**Figure S12: Locations in which cobavirus hosts were found based on a 16S rRNA survey in the NR Blast database and in the Tara Ocean samples.**

# Publication bibliography

Aird, Daniel; Ross, Michael G.; Chen, Wei-Sheng; Danielsson, Maxwell; Fennell, Timothy; Russ, Carsten et al. (2011): Analyzing and minimizing PCR amplification bias in Illumina sequencing libraries. In *Genome biology* 12 (2), R18. DOI: 10.1186/gb-2011-12-2-r18.

Angly, Florent; Youle, Merry; Nosrat, Bahador; Srinagesh, Shailaja; Rodriguez-Brito, Beltran; McNairnie, Patrick et al. (2009): Genomic analysis of multiple Roseophage SIO1 strains. In *Environmental microbiology* 11 (11), pp. 2863–2873. DOI: 10.1111/j.1462-2920.2009.02021.x.

Balch, W. E.; Fox, G. E.; Magrum, L. J.; Woese, C. R.; Wolfe, R. S. (1979): Methanogens: reevaluation of a unique biological group. In *Microbiological Reviews* 43 (2), pp. 260–296.

Bankevich, Anton; Nurk, Sergey; Antipov, Dmitry; Gurevich, Alexey A.; Dvorkin, Mikhail; Kulikov, Alexander S. et al. (2012): SPAdes: A New Genome Assembly Algorithm and Its Applications to Single-Cell Sequencing. In *Journal of Computational Biology* 19 (5), pp. 455–477. DOI: 10.1089/cmb.2012.0021.

Bolger, Anthony M.; Lohse, Marc; Usadel, Bjoern (2014): Trimmomatic: a flexible trimmer for Illumina sequence data. In *Bioinformatics (Oxford, England)* 30 (15), pp. 2114–2120. DOI: 10.1093/bioinformatics/btu170.

Brum, Jennifer R.; Ignacio-Espinoza, J. Cesar; Roux, Simon; Doulcier, Guilhem; Acinas, Silvia G.; Alberti, Adriana et al. (2015): Patterns and ecological drivers of ocean viral communities. In *Science* 348. DOI: 10.1126/science.1261498.

García-Alcalde, Fernando; Okonechnikov, Konstantin; Carbonell, José; Cruz, Luis M.; Götz, Stefan; Tarazona, Sonia et al. (2012): Qualimap: evaluating next-generation sequencing alignment data. In *Bioinformatics (Oxford, England)* 28 (20), pp. 2678–2679. DOI: 10.1093/bioinformatics/bts503.

Illumina, Inc (2015): Nextera XT Library Prep: Tips and Troubleshooting. Available online at https://support.illumina.com/content/dam/illumina-support/documents/documentation/chemistry_documentation/samplepreps_nextera/nextera-xt/nextera-xt-troubleshooting-guide.pdf.

Merrill, Bryan D.; Ward, Andy T.; Grose, Julianne H.; Hope, Sandra (2016): Software-based analysis of bacteriophage genomes, physical ends, and packaging strategies. In *BMC genomics* 17, p. 679. DOI: 10.1186/s12864-016-3018-2.

Nishimura, Yosuke; Watai, Hiroyasu; Honda, Takashi; Mihara, Tomoko; Omae, Kimiho; Roux, Simon et al. (2017): Environmental viral genomes shed new light on virus-host interactions in the ocean. In *mSphere* 2 (2). DOI: 10.1128/mSphere.00359-16.

Paez-Espino, David; Chen, I-Min A.; Palaniappan, Krishna; Ratner, Anna; Chu, Ken; Szeto, Ernest et al. (2017): IMG/VR. A database of cultured and uncultured DNA Viruses and retroviruses. In *Nucleic acids research* 45 (D1), pp. D457-D465. DOI: 10.1093/nar/gkw1030.

Paez-Espino, David; Eloe-Fadrosh, Emiley A.; Pavlopoulos, Georgios A.; Thomas, Alex D.; Huntemann, Marcel; Mikhailova, Natalia et al. (2016): Uncovering Earth's virome. In *Nature* 536 (7617), pp. 425–430. DOI: 10.1038/nature19094.

Robinson, James T.; Thorvaldsdóttir, Helga; Winckler, Wendy; Guttman, Mitchell; Lander, Eric S.; Getz, Gad; Mesirov, Jill P. (2011): Integrative genomics viewer. In *Nature Biotechnology* 29. DOI: 10.1038/nbt.1754.

Roux, Simon; Brum, Jennifer R.; Dutilh, Bas E.; Sunagawa, Shinichi; Duhaime, Melissa B.; Loy, Alexander et al. (2016): Ecogenomics and potential biogeochemical impacts of globally abundant ocean viruses. In *Nature* 537 (7622), pp. 689–693. DOI: 10.1038/nature19366.
